# A Farm-to-Fork Quantitative Microbial Exposure Assessment of β-Lactam-Resistant *Escherichia coli* among U.S. Beef Consumers

**DOI:** 10.3390/microorganisms10030661

**Published:** 2022-03-19

**Authors:** Yangjunna Zhang, John W. Schmidt, Terrance M. Arthur, Tommy L. Wheeler, Qi Zhang, Bing Wang

**Affiliations:** 1 Institute of Food Science and Engineering, Hangzhou Medical College, Hangzhou 310013, China; yzhan241@hmc.edu.cn; 2 United States Department of Agriculture, Agricultural Research Service, Roman L. Hruska U.S. Meat Animal Research Center, Clay Center, NE 68933, USA; john.w.schmidt@usda.gov (J.W.S.); terrance.arthur@usda.gov (T.M.A.); tommy.wheeler@usda.gov (T.L.W.); 3 Department of Mathematics and Statistics, College of Engineering and Physical Sciences, University of New Hampshire, Durham, NH 03824, USA; qi.zhang2@unh.edu; 4 Department of Food Science and Technology, College of Agricultural Sciences and Natural Resources, University of Nebraska-Lincoln, Lincoln, NE 68588, USA

**Keywords:** simulation model, antibiotic resistance, beef consumption, risk assessment

## Abstract

Integrated quantitative descriptions of the transmission of β-lactam-resistant *Escherichia coli* (BR-EC) from commercial beef products to consumers are not available. Here, a quantitative microbial exposure assessment model was established to simulate the fate of BR-EC in a farm-to-fork continuum and provide an estimate of BR-EC exposure among beef consumers in the U.S. The model compared the per-serving exposures from the consumption of intact beef cuts, non-intact beef cuts, and ground beef. Additionally, scenario analysis was performed to evaluate the relative contribution of antibiotic use during beef cattle production to the level of human exposure to BR-EC. The model predicted mean numbers of BR-EC of 1.7 × 10^−4^, 8.7 × 10^−4^, and 6.9 × 10^−1^ CFU/serving for intact beef cuts, non-intact beef cuts, and ground beef, respectively, at the time of consumption. Sensitivity analyses using the baseline model suggested that factors related to sectors along the supply chain, i.e., feedlots, processing plants, retailers, and consumers, were all important for controlling human exposure to BR-EC. Interventions at the processing and post-processing stages are expected to be most effective. Simulation results showed that a decrease in antibiotic use among beef cattle might be associated with a reduction in exposure to BR-EC from beef consumption. However, the absolute reduction was moderate, indicating that the effectiveness of restricting antibiotic use as a standalone strategy for mitigating human exposure to BR-EC through beef consumption is still uncertain. Good cooking and hygiene practices at home and advanced safety management practices in the beef processing and post-processing continuum are more powerful approaches for reducing human exposure to antibiotic-resistant bacteria in beef products.

## 1. Introduction

Meat products are considered potential vehicles for the delivery of antibiotic-resistant bacteria (ARB) to humans through food consumption [1]. ARB harbored in and on livestock animals and production environments may be transferred to the carcass at harvest, persist through interventions during meat processing, and eventually end up on consumers’ plates. Various environmental and operational factors may influence the dynamics of ARB during the long transmission chain from primary production to human exposure. This complexity makes it challenging to disentangle the transmission mechanisms and identify effective mitigation strategies.

In recent decades, the association of *Escherichia coli* (*E. coli*) with antibiotic resistance has drawn increasing attention worldwide. *E. coli* is widely used as a sentinel microorganism for monitoring resistant microbial contamination in food and the environment, including beef production and processing [2,3]. β-Lactam antibiotics that are critically important to human medicine (e.g., ampicillin, penicillins, cephalosporins, carbapenems) have long been used to prevent/treat diseases in cattle [4,5]. For instance, in the U.S., third-generation cephalosporins are approved for the treatment and control of diseases in cattle such as bovine respiratory disease, metritis, foot rot, and mastitis [6]. Pre-harvest use of antibiotics may contribute to the development of bacterial β-lactam resistance in live animals, and processing and post-processing steps may affect the transmission of contamination to the final meat products. β-Lactam-resistant *E. coli* (BR-EC) have been detected in various cattle-related samples, including cattle intestines/feces, hides/carcasses, and beef products at retail markets [7,8,9]. As a result, BR-EC intake through the consumption of contaminated meat meals is likely and has the potential to compromise the treatment efficacy of those medically important antibiotics when foodborne infections occur [10].

Although the generic *E. coli* examined in antibiotic resistance monitoring studies are typically commensal and non-pathogenic, generalized concerns remain because commensal BR-EC may transmit (by horizontal gene transfer) antibiotic resistance genes (ARGs) to pathogens in the human gastrointestinal system following consumption [3,11,12]. However, the presence of an ARG in commensal *E. coli* should not be overinterpreted, as the rate of ARG transfer between commensal and pathogenic strains *in situ* is difficult to accurately measure. Hence, instead of focusing on quantifying the dose–response relationship between BR-EC ingestion and the risk of human infection considering β-lactam resistance transfer, it would be more useful and practical to evaluate the intake level of BR-EC at the time of food consumption as a crude indicator or sentinel of public health concerns.

In contrast to the considerable body of work characterizing BR-EC at various steps in beef production and processing, attempts to quantitatively synthesize the relevant evidence for a systems assessment of BR-EC transmission from beef production to consumption are limited. Quantitative microbial exposure assessment (QMEA) is a widely endorsed approach for microbial food safety management, and here a QMEA model was established to simulate the transmission of BR-EC in the U.S. farm-to-fork beef continuum using BR-EC ingested via one serving of beef as the output of interest. The developed model was used to (1) compare the levels of BR-EC ingested via the consumption of various beef products, i.e., intact and non-intact beef cuts and ground beef; and (2) identify significant factors in the beef production, processing, and preparation continuum for controlling human exposure to BR-EC. The findings may be used to support science-based recommendations for identifying candidate steps for intervention implementation and further optimizing antibiotic resistance mitigation strategies.

## 2. Materials and Methods

### 2.1. Model Overview

**Figure 1:**
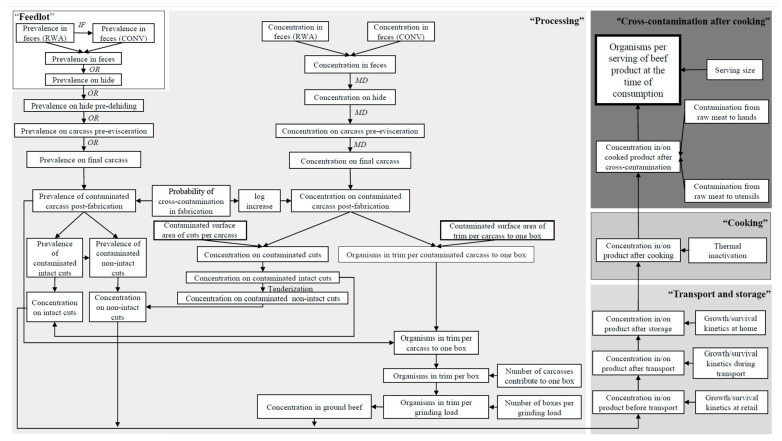
Schematic diagram of the quantitative microbial exposure assessment model. RWA—raised without antibiotics; CONV—conventional; *IF*—impact factor; *OR*—odds ratio; *MD*—mean difference.

The farm-to-fork QMEA model simulated the passage of BR-EC by quantifying the changes in microbial prevalence and concentration at various steps along the beef supply chain. The completed model comprised five consecutive modules: “feedlot”, “processing”, “transport and storage”, “cooking”, and “cross-contamination after cooking”. The final model outputs were the microbial loads of BR-EC in one serving of intact beef cuts, non-intact beef cuts, and ground beef at the time of consumption. The schematic diagram upon which the QMEA model was developed is illustrated in Figure 1. To capture the variability and uncertainty of the stochastic estimates of model outputs, a one-dimensional Monte Carlo simulation using Latin hypercube sampling for 100,000 iterations was performed in Microsoft Excel^®^ 2013 (Microsoft Corp., Redmond, WA, USA) with the add-on software @Risk^®^ 7.5 (Palisade Corp., Ithaca, NY, USA).

### 2.2. Exposure Assessment

The fate of BR-EC was simulated and quantified from cattle feces to hides, from hides to carcasses, through processing steps at the slaughterhouse, transport from the processing plant to retail, retail storage, transport from retail to home, home storage, food handling and preparation, and ultimately to consumption.

Along the chain, the prevalence change was quantified by the odds ratio (*OR*), which links the prevalence before and after a particular step, using Equation (1) as a rearrangement of the calculation of *OR* following epidemiological concepts [13]:

(1) $$P_{i+1}=\frac{OR\times P_{i}}{1-P_{i}+OR\times P_{i}\;}$$

where *P_i_* and *P*_*i*+1_ are the prevalences before and after a step, respectively. An *OR* value greater than 1 indicates an increase in prevalence, whereas a value less than 1 implies a decrease.

The concentration change was quantified by the mean difference (*MD*), which links the concentration before and after a particular step, using Equation (2):

(2) $$C_{i+1}=C_{i}-MD$$

where *C_i_* and *C*_*i*+1_ are the concentrations in log_10_ CFU/unit before and after a particular step among enumerable samples. A positive *MD* indicates a decrease in microbial load, while a negative value indicates an increase.

*OR* and *MD* were the “joints” of the stochastic QMEA model connecting *E. coli* contamination step by step. The incorporation of *OR* and *MD* simplified the prediction of contamination caused by a specific step by avoiding the need for simulations of the complex mechanisms within the step. Three specific strategies were adopted to minimize the possibility that this simplification did not capture the full variance. First, instead of relying on data identified sporadically or by convenience, the estimation of *OR*s and *MD*s at various steps was based on a comprehensive literature review, and their probability distributions were parameterized using the synthesized results of a random-effects meta-analysis (MA). Second, when transferring the MA results into model input distributions, the variances of *OR* and *MD* were estimated by considering both within-study variance due to sampling errors and between-study heterogeneity. Last, to avoid unrealistic values in the probabilistic distributions of *OR* and *MD*, a wider range was applied in the truncation technique by setting the minimum lower limit and the maximum upper limit of the 95% confidence intervals of *ORs* and *MDs* estimated in primary studies as the boundaries. This truncation approach permits the capture of the most extreme observations in documented primary studies and thus covers an even wider range than the 95% prediction interval of the aggregate effect size, thereby maximizing the capability of capturing a representative variation to offset the effect of the simplified model structure. Details of the data collection and synthesis for *OR* and *MD* estimation are provided in the Supplementary Materials, Text S1.

#### 2.2.1. Feedlot

The feedlot module started with the percentage of cattle raised with antibiotics in the U.S., which was fixed at 90.1% (*P_CONV_*) based on the most recent data estimating the percentage of commercial cattle raised on conventional feedlots (CONV) where antibiotics are allowed [14]. The remaining 9.9% were hypothesized to be raised without antibiotics (RWA). The term “RWA” was used as a contrast to “CONV” and indicates that no antibiotics of any kind were used in animal husbandry for any purposes, including therapeutic or prophylactic use. Subsequent model inputs were estimated separately for CONV and RWA systems if possible, as summarized in Table S1.

##### Prevalence of BR-EC in Feces at the Feedlot

The estimation of fecal BR-EC prevalence at the feedlot started with that of the RWA system, which was subsequently related to CONV animals using an impact factor (*IF*, essentially an *OR*) to indicate the impact of feedlot antibiotic use on BR-EC shedding. To capture seasonal effects on *IF*, a hierarchical model with a beta-binomial mixture distribution was developed by using the maximum likelihood estimation method to estimate the prevalence of BR-EC in RWA cattle feces. Values of prevalence estimates together with sample sizes were extracted from relevant primary studies and fit to a beta-binomial mixture distribution accounting for inter-study variation and seasonal differences (where applicable). The fitting process was run in R. 3.4.0, as detailed in the Supplementary Materials, Text S2. The estimate of *IF* was used to translate BR-EC prevalence at CONV feedlots to BR-EC prevalence at RWA feedlots using Equation (1). *IF* was estimated using the same process described for *OR* in the Supplementary Materials, Text S1.

##### Prevalence of BR-EC on Hides at the Feedlot

An *OR* was incorporated to link the prevalence of BR-EC in feces to that on hides by using Equation (1). The estimation of *OR* distribution parameters was based on data extracted from studies reporting changes in *E. coli* species regardless of antibiotic susceptibility profile or pathogenicity, as the dissemination behaviors of different *E. coli* strains were assumed to be the same.

#### 2.2.2. Processing

The processing module simulated the changes in BR-EC through primary and secondary processing procedures, beginning at the time that the cattle were stunned at the slaughterhouse and ending with packaged servings of three categories of beef products: intact beef cuts, non-intact beef cuts, and ground beef. Tables S2–S6 summarize the variables covered in this module.

##### Composition Variables of Beef Production

The characteristics of intermediate and end beef products summarized in Table S2 are referred to as composition variables of beef production. The carcass weight (*W_carc_*) was described using four independent normal distributions representing beef produced from different types of U.S. cattle: steer, heifer, cow, and bull. In a particular iteration, the distributions of *W_carc_*, the fraction of carcass ending up as cuts/trim (*F_cuts_carc_* or *F_trim_carc_*), and the total surface area (*TSA*) were dependent on the particular type of cattle being simulated. The carcass surface is generally considered the major source of contamination of beef products [15], but the area of the surface contaminated with *E. coli* (*TCA*), especially BR-EC, remains unclear [16]. This uncertainty was considered in the estimation of this variable, which had a possible range from a minimum area of 30 cm^2^ according to the measurable detection threshold to a maximum area equal to the total carcass surface area (*TSA*), which varies by cattle type.

##### BR-EC Contamination on Hides before Dehiding

To estimate the prevalence of BR-EC on hides at the processing plant, an *OR* (*OR_hh_ farm_plant_*) describing the difference in *E. coli* prevalence on the hides of animals at the feedlot versus the processing plant was estimated and incorporated using Equation (1) (Table S3).

Concentration data were incorporated in the model from this point, as no concentration data are available separately for RWA and CONV animals prior to dehiding. The concentration on hides was computed from that in feces, assuming a fecal–hide transmission route. The bacterial concentration in cecal contents (log_10_ CFU/g) at a commercial processing plant reported by Vikram et al. [17] was used as a surrogate for the concentration of *E. coli* in feces before dehiding at either RWA or CONV feedlots (Table S3). This is a reasonable substitution, as the processing interventions were assumed to have minimal impact on the levels of microorganisms in cattle colons. To estimate the bacterial concentration on hides from the bacterial concentration in feces, the *MD* (*MD_fh_BR_plant_*) was derived to build the relationship between the concentrations in feces and on hides. A normal distribution of the logarithmic *MD* was fit via the MA approach as described in Text S1 by using microbial load data for third-generation cephalosporin-resistant *E. coli* in fecal and on-hide samples from a processing plant, as reported by Schmidt et al. [8].

##### Primary Processing of Beef Carcasses

The simulation of the primary processing mainly covered the carcass dressing steps: dehiding, evisceration, splitting of carcasses, and the final carcass after chilling. For each of these steps, *OR*s and *MD*s were estimated for the changes in prevalence and concentration according to the MA approach as described in Text S1 by using the data extracted from multiple relevant studies listed in Table S3. As mentioned above, for a particular step, the *OR*s or *MD*s were estimated based on data without differentiation of antibiotic susceptibility profiles, as our previous study showed that processing steps and interventions have similar effects on susceptible and resistant strains [18].

**From pre-dehiding to pre-evisceration.** This step covers the dehiding process and the washing interventions performed immediately afterward. The changes in contamination, quantified as *OR* and *MD* in this step, therefore represent a mixed effect of physical removal, cross-contamination, and decontamination due to the generic interventions commonly applied in beef processing plants in the U.S. *OR* at this step (*OR_hc_hide_carc_*) was estimated by considering the difference between the high- and low-shedding seasons based on multiple relevant studies evaluating *E. coli* contamination on hide samples collected at processing plants and on carcasses before evisceration (Table S3). It was assumed that the prevalence changes on the hides of RWA and CONV cattle did not differ between resistant and susceptible strains. To estimate the *MD* (*MD_hc_BR_hide_carc_*) for concentration changes, concentrations of BR-EC on hides and on pre-evisceration carcasses from an empirical study that followed up the same cohorts of animals at a processing plant were used [8].

**From pre-evisceration to final carcass.** This step covers evisceration and splitting of the carcass, chemical/physical interventions such as carcass washing with dilute organic acid/hot water/steam immediately after these steps, and overnight chilling of the carcass within 24 h of evisceration. Studies have shown that intestinal rupture during evisceration has a limited impact on carcass contamination [19]. Therefore, contamination on the final carcass was assumed to be solely from contamination on the pre-evisceration carcass. A uniform microbial distribution on the beef carcass surface was assumed.

The data used to estimate the *OR* (*OR_cc_preevis_final_*) of BR-EC at this step were extracted from two biomapping studies that reported the prevalence of *E. coli* on the carcass surface sampled pre-evisceration and on the final carcass surface after a chilling process [8,20]. The *MD* in BR-EC (*MD_cc_BR_preevis_final_*) at this step could not be quantified via the MA approach and was estimated by an adjusted deterministic value (Table S3), based on the very low levels detected [8].

##### Secondary Processing of Beef Products

**Processing of intact beef cuts.** After entering the fabrication area, an individual final carcass is usually cut into five primal cuts of chuck, rib, loin, round, and shank, which are further partitioned into smaller portions called sub-primal cuts. The same types of primal and sub-primals are usually processed on the same line.

In the absence of data, the probability of cross-contamination during fabrication was assumed to be a uniform distribution ranging between 0 and 1. The USDA-FSIS (2001) estimate of the population increase of *E. coli* O157:H7 during fabrication (fit to Pert distributions in Table S4**)** was used as a substitute for the changes in BR-EC [16]. To estimate the concentration of BR-EC on a beef cut (*C_N_BR_int_*), four scenarios of the occurrence of cross-contamination and the presence or absence of contamination on the carcasses that the beef cuts were derived from were considered. The concentrations were first calculated as CFU/100 cm^2^ of contaminated carcass surface destined to a piece of beef cut (*C_c_BR_postfabr_*), which was then transformed into CFU/g of meat. The calculations are listed in Tables S4 and S5.

**Processing of non-intact beef cuts (tenderization).** Non-intact beef cuts are intact beef cuts that have been injected or enhanced with marinade, flavoring, or tenderizing solutions or mechanically tenderized by needling, blading, cubing, or pounding devices [21]. The input variables and calculations for BR-EC changes on non-intact beef cuts due to tenderization are summarized in Table S5. Because of a lack of information, the probability of lateral contamination occurrence due to sharing tenderization equipment between cuts (*P_lat_cntm_*) was assumed to follow a uniform distribution ranging between 0 and 1. To describe the quantity of bacteria transferred between cuts, data from a study that quantified the amount of *E. coli* O157:H7 transferred from a surface-contaminated beef cut to four other pieces of sterilized beef cuts via blade tenderization were used [22]. The cited study showed that the concentrations of *E. coli* O157:H7 recovered from the second, third, fourth, and fifth pieces were reduced from the first contaminated piece by approximately 0.5, 1.0, 1.5, and 1.5 log_10_ CFU/g, respectively. Hence, it was assumed that lateral contamination could cause a 0–1.5 log decrease in BR-EC in a piece of a contaminated beef cut but a 0–1.5 log increase in a piece of non-contaminated beef cut before tenderization.

**Processing of ground beef.** Ground beef processing starts from trim. Trim is obtained as the byproduct at the end of each line by cutting excess fat and lean off the primal and sub-primal cuts. At the end of each line, trim is loaded into one combo bin, which is defined as a container that can hold approximately 907 kg (2000 lb) of trim (*W_trim_bin_*) [16] (Table S6). Without line-specific information, the trim harvested from different lines was assumed to have the same level of *E. coli* contamination. Ground beef is produced by mixing and grinding the five bins of trim in a grinding load (*b*) that can hold approximately 4536 kg (10,000 lb) of trim [16] (Table S2). Given the trim weight per bin (*W_trim_bin_*), the number of carcasses contributing trim to each bin (*c*) can be estimated with variation due to varying chilled carcass weights. After mixing and grinding, the ground beef is partitioned into servings before transport from the processing plant to retail stores. *Escherichia coli* was assumed to be homogeneously distributed in the ground beef.

The input variables used to calculate the concentration of BR-EC in ground beef are summarized in Tables S2 and S6 and follow the model that we previously developed for *Salmonella* in ground pork [23]. Similar to intact beef cuts, contamination on trim is from the contaminated carcass surface. From a final carcass, 75% of the total surface area ends in trim (*F_trim_area_*), one-fifth of which contributes to one bin (*A_trim_carc_bin_*). The number of BR-EC on this portion of the surface area (*N_BR_trim_carc_bin_*) was determined by *C_c_BR_postfabr_* and the contaminated area of this portion (*TCA_trim_bin_*). The total number of organisms in one bin (*N_BR_bin_*) was calculated by multiplying the sum of BR-EC organisms in trim per carcass by the number of carcasses *c* in one bin, and the number of organisms in one grinding load was the sum of bacteria in five bins. Finally, the concentration of BR-EC was calculated by dividing the number of organisms per grinding load by the weight of the grinding load, assuming a homogeneous distribution of BR-EC in contaminated grind loads.

#### 2.2.3. Transport and Storage

The transport and storage module simulated the fate of BR-EC contamination of beef products from processing plant to retail, at retail, from retail to home, and then at home. The change in contamination was quantified on/in the three types of beef products based on the environmental conditions at each step (Table S7). Products were considered to be transported or stored as either “frozen” or “fresh”. Exclusively in this study, “frozen” products were defined as beef meat stored at temperatures below 0 °C, while “fresh” products were defined as beef meat stored at temperatures of 0 °C or higher. Please note that the terms “frozen” and “fresh” were used in this study to describe the products’ status based on the impact of temperature on bacterial behavior and did not follow regulatory definitions for labeling purposes.

The minimum temperature for quantifying *E. coli* growth in meat is reported to be 10.08 °C (*T_obs_*) [24]. Hence, the population change of *E. coli* was only considered as growth when the storage/transport temperature was greater than *T_obs_*, with no changes in “frozen” products or fresh products at temperatures between 0 and *T_obs_*. Equations and parameters for the growth kinetics model of *E. coli* were adopted from Baranyi and Roberts [25], where *r_max_* (maximum specific growth rate in ln CFU/h) and *λ* (lag phase duration in hours) were calculated using Equations (3) and (4) for beef cuts and Equations (5) and (6) for ground beef, respectively:

(3) $$r_{max}=\frac{{\left(0.02\times T+0.031\right)}^{2}}{\ln10}$$

(4) $$\lambda ={\;e}^{-0.121\times T+5.147}$$

(5) $$r_{max}=\frac{{\left(0.028\times \left(T-3.7942\right)\times \left(1-e^{0.7524\times \left(T-47.1646\right)}\right)\right)}^{2}}{\ln10}$$

(6) $$\lambda =-1.033+14.957\times e^{-\frac{\ln2}{\ln\left(11.253^{2}\right)}\times \ln{\left(\frac{\left(T-10.641\right)\times \left(11.253^{2}-1\right)}{6.376\times 11.253}+1\right)}^{2}}$$

##### Transport from the Processing Plant to Retail

Cold chain transportation was considered in this study for the transport of beef products from the processing plant to retail, as this represents common practice in the U.S. Overall, the internal temperature of meat can be controlled at less than 7 °C in the cabinet [26]. Jakubowski monitored the air temperature inside the cooling chamber of three vehicles carrying meat products for a food business company following different transport routes and showed that the average temperature inside the refrigerated body varied between 5.4 and 6.5 °C, which can efficiently minimize bacterial spoilage [27]. Short-term temperatures exceeding 7 °C were expected due to temporary chamber door opening for loading and unloading, but the effects of these transient increases in temperature on microbial growth can be considered negligible. As a result, no organism growth was modeled during the transport of beef products from the processing plant to the retail store.

##### Retail Storage

In 2005, it was estimated that 90.8% and 9.2% of beef meat sold in Canada was stored in refrigerators (“fresh” status) and freezers (“frozen” status), respectively [19]. As approximately 8.9% of beef products are stored in retail refrigerators with temperatures below 0 °C [28], the percentages of “fresh” and “frozen” meat at retail were adjusted to 82.7% and 17.3%, respectively, for use as substitute data for the U.S. The refrigeration temperature at retail (*T_retail_*) was fit to a Laplace distribution using survey data with the minimum value truncated at 0 [28]. The most likely storage time of beef meat at retail (*Time_retail_*) was reported to be between 0.5 and 1.5 days, with minimum and maximum values of 0 and 14 days [16,29].

##### Transport from Retail to Home

Beef products sold as “frozen” were assumed to have a transport temperature below 0 °C, whereas beef products sold as “fresh” were assumed to undergo a temperature increase during transport. The only data available are internal temperatures of beef products at the time of arrival at consumers’ homes [28], which were therefore used as a surrogate for temperature during transport (*T_trans_*). Transport time (*Time_trans_*) data were obtained from the same source (Table S7).

##### Home Storage

Several home storage situations were considered based on the status of products sold at retail and storage conditions at home. Beef products sold and transported as “frozen” were assumed to be stored in the freezer at home, and hence no bacterial growth was modeled in this situation. For products sold and transported as “fresh”, the number of bacteria remained unchanged when the products were stored in the freezer at home. For beef meat sold, transported, and stored as “fresh”, the temperature (*T_home_*) at home was determined by a cumulative empirical distribution built from data obtained by EcoSure [28]. The refrigerator storage times of beef cuts (*Time_home_*) and ground beef (*Time_gb_home_*) were each fit to a cumulative distribution with several time intervals and their corresponding cumulative probabilities [30].

#### 2.2.4. Cooking

As shown in Table S8, the thermal inactivation kinetics of BR-EC on beef cuts and in ground beef were simulated using models from another risk assessment study of *E. coli* O157:H7 [19]. Briefly, data for each beef product were fit to a linear regression by plotting the average measured log reduction against internal meat temperature (beef cuts: 48.9–76.7 °C; ground beef: 56.1–74.4 °C), regardless of cooking method or beef thickness. The internal temperatures of beef cuts and ground beef during cooking were fit separately to stochastic distributions using data from EcoSure [28].

#### 2.2.5. Cross-Contamination after Cooking

As shown in Table S9, two cross-contamination routes were studied in this module: via hands or kitchen utensils contaminated by contacting raw meat. The transmission of bacteria via these two routes was assumed to occur independently. To quantify the cross-contamination mechanism, data on the transfer rate of surrogate bacteria from raw chicken to hands/utensils after cooking and then to cooked meals summarized by Smadi and Sargeant [31] were used as surrogate data for beef preparation in this model.

### 2.3. Exposure Estimates of the Baseline Model

The baseline model simulated the situation of cattle management, beef processing, transport, storage, preparation, and handling under current common conditions in the U.S. as described in Section 2.2 and Tables S1–S9. The number of BR-EC organisms per serving of intact/non-intact beef cut/ground beef at the time of consumption served as the output of this model, since there are no documented dose–response models for oral ingestion of BR-EC. Relevant calculations are listed in Table S8. Serving size was quantified based on the recommended portion size for the general adult population and/or commonly served portion sizes [32,33]. A portion size of 85 g (3 ounces) was chosen for ground beef, and a portion size of 227 g (8 ounces) was used for beef cuts.

Model validation is usually performed by comparing the model’s prediction with the status in reality to evaluate the model’s precision and reliability [34]. Instead of using all three types of beef products to validate the model, ground beef was chosen as representative, as its model structure is the same as that of other beef products except for the extra processing steps, and ground beef is of greatest food safety concern. However, due to the lack of empirical data on BR-EC contamination in cooked beef meals [35], direct validation is practically impossible. Therefore, an alternative approach, i.e., comparisons of predictions of different models, was followed based on the suggestions in FAO and WHO’s microbiological risk assessment guidance for food [36]. Two published retail-to-consumption exposure assessments [35,37] predicting the population of BR-EC in cooked ground beef meals were relevant and selected for comparison with our model outputs.

### 2.4. What-If Scenario Analysis

Compared with the baseline (*P_CONV_* = 90.1%), the value of *P_CONV_* was varied between 0 and 100% to simulate the effects of changing the proportion of cattle administered antibiotics in the U.S. on BR-EC exposure through beef consumption. There was no attempt in this study to evaluate the impact of changes in specific antibiotic regimens (such as dose, duration, or routes of β-lactam or other classes of drugs) on human exposure due to data limitations.

### 2.5. Sensitivity Analysis

Sensitivity analysis was performed on the baseline model to identify the most important stochastic input variables affecting the model output. The impact of the input was evaluated by calculating the absolute change in the output mean caused by varying the input’s value. To evaluate a specific input, 100,000 simulated data for the input from the baseline were grouped into 20 bins with 5000 data in each, ranging from the input’s lowest to highest values. The output mean was calculated for each bin of the target input. The difference between the maximum and minimum values of the 20 output means indicated the input’s impact on the output mean. These steps were repeated for all stochastic input variables. To generate a combined display of the results of the sensitivity analyses for all 6 combinations of microorganisms and beef products (BR-EC in three beef products), the ranks of all of the inputs’ impacts were rescaled within a range from 0 (smallest absolute change) to 1 (largest absolute change) and displayed in heat map charts, with deeper color denoting a stronger impact of varying the inputs on model output change.

## 3. Results and Discussion

### 3.1. Baseline Model Estimates and Validation

Under the baseline conditions, the estimated average number of BR-EC was 1.7 × 10^−4^ (95% CI: 1.1 × 10^−4^–2.4 × 10^−4^), 8.7 × 10^−4^ (95% CI: 4.1 × 10^−4^–1.3 × 10^−3^), and 6.9 × 10^−1^ (95% CI: 0–1.7) per serving of intact beef cuts, non-intact beef cuts, and ground beef, respectively.

Documented empirical data on the observed number of BR-EC in a cooked beef meal are limited; therefore, predictive values of BR-EC in ground beef from two published exposure assessments were used for comparison. Evers et al. predicted that the average number of BR-EC was 0.275 CFU at consumption for 75 g of non-specified beef meat [37], which can be adjusted to 3.1 × 10^−1^ CFU per serving (85 g) of ground beef to be comparable to this study, within an order of magnitude. Nekouei et al. reported that the concentration of ceftriaxone-resistant *E. coli* in ground beef after cooking was between −6 and −4 log_10_ CFU/g [35], which can be translated to 8.5 × 10^−5^ to 8.5 × 10^−3^ CFU/serving in this study. Unlike the estimates by Evers et al. [37], our model covers the display period at retail and transport of ground beef from retail to home, which are included due to the possibility of bacterial growth and might explain the higher final estimate in our model. Our estimate is also higher than that given by Nekouei et al.’s model, as the latter did not include cross-contamination during food preparation and only focused on the resistance to one specific antibiotic drug in the β-lactam group [35]. Considering the variation across studies, our model’s estimate of BR-EC contamination in ground beef is acceptable with respect to current beef supply systems.

**Figure 2:**
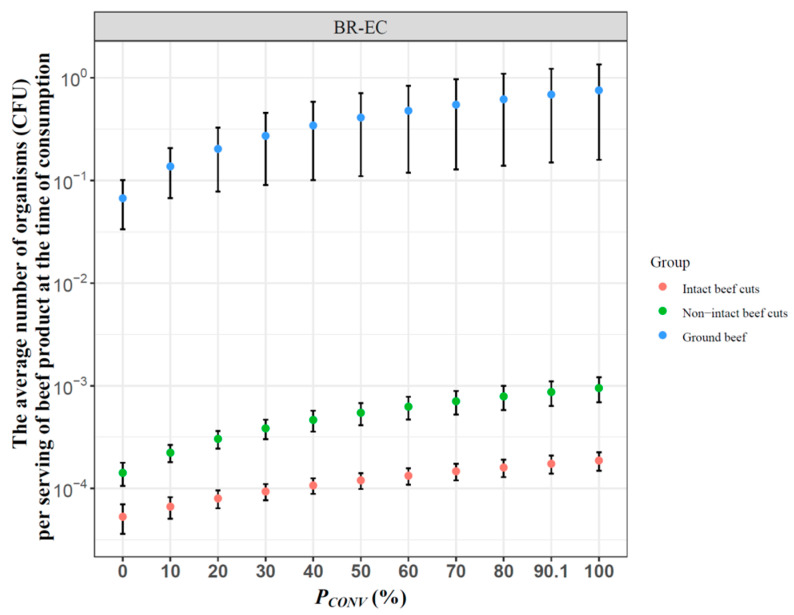
Impacts of antibiotic use in U.S. beef cattle agrisystems on the average number (± standard error) of BR-EC per serving of intact beef cuts, non-intact beef cuts, and ground beef (CFU/serving) at the time of consumption. Baseline: *P_CONV_* = 90.1%, referring to the percentage of cattle raised with antibiotics in the U.S.

Based on our simulation, the number of BR-EC was expected to generally be highest in ground beef, followed by non-intact and then intact beef cuts (Figure 2). Smith et al. showed a similar trend for pathogenic *E. coli* [19]. From 2003 to 2012, 22 outbreaks of *E. coli* O157:H7 reported in the U.S. were associated with beef, of which 17 were attributed to the consumption of ground beef [38]. The major reason for this higher risk is that ground beef is more likely to include contaminated tissues due to the commingling of trim from multiple carcasses into one grinding load, whereas a piece of beef cut comes from a single carcass. In addition, *E. coli* organisms may be transferred from contaminated regions to uncontaminated regions during mixing and grinding. Furthermore, the organisms present inside the ground meat may be protected by fat tissue during the thermal inactivation process [39]. Although the occurrence of *E. coli* contamination in non-intact beef cuts was lower than that in ground beef, the tenderization of non-intact beef cuts not only increases the probability of cross-contamination among beef cuts, knives, gloves, hands, and other equipment but may also introduce surface organisms into deep, internal tissues [40].

### 3.2. Effect of Antibiotic Use on Human Exposure to BR-EC via Consumption of Beef Meat

Alternative scenarios were analyzed to evaluate the changes in human exposure to BR-EC through consumption of beef meals as a result of changes in the proportion of CONV cattle (*P_CONV_*) in the U.S. from 0 to 100%, with 90.1% as the baseline. It was predicted that full removal of antibiotic use at the feedlot would not lead to zero human exposure to BR-EC through beef consumption (*P_CONV_* = 0% in Figure 2). The major reason is that even without or with limited use of antibiotics, resistant bacteria are still readily detected, including in RWA animals [9,17]. Antibiotic resistance is an ancient and ubiquitous phenomenon that occurs in essentially any environment with bacterial populations [41,42], including very extreme cases such as remote polar regions with no history of animal husbandry [43]. Although there are no empirical data endorsing the presence of antibiotic-resistant *E. coli* in cooked beef meals originating from RWA systems, antibiotic-resistant *E. coli* are frequently detected in retail RWA beef meat [9,44,45]. In agreement with previous studies, our results demonstrate that both primary production systems, CONV and RWA, can allow antibiotic-resistant *E. coli* to enter the food chain.

As shown in Figure 2, as *P_CONV_* was reduced from 100 to 0%, indicating a change from antibiotic administration to all beef cattle in the U.S. to none, the average number of BR-EC ingested decreased gradually from 1.9 × 10^−4^ to 5.3 × 10^−5^ for intact beef cuts, 9.5 × 10^−4^ to 1.4 × 10^−4^ for non-intact beef cuts, and 7.6 × 10^−1^ to 6.7 × 10^−2^ CFU/serving for ground beef at the time of consumption. A decrease in the level of exposure to BR-EC with increasingly restricted antibiotic use at the feedlot was expected, although the magnitude of this decrease was not large.

To investigate the reason for this moderate decrease, the differences in the level of BR-EC contamination between CONV and RWA systems along the beef processing chain were examined. Four variables in the “processing” module (Table S3) were set as intermediate outputs for comparison purposes: the concentrations of BR-EC in cattle feces at the processing plant (*C_f_BR_RWA/CONV_*), on hides at the processing plant (*C_h_BR_plant_RWA/CONV_*), on pre-evisceration carcasses (*C_c_BR_preevis_RWA/CONV_*), and on final carcasses (*C_c_BR_final_RWA/CONV_*). In the baseline condition, where *P_CONV_* was estimated as 90.1%, the difference in the mean BR-EC concentration at these steps between RWA and CONV animals gradually decreased from 0.33 CFU/g in cattle feces to 0.00078 CFU/100 cm^2^ on the final carcass. These differences would shrink further if smaller values of *P_CONV_* were used. The highly efficient bacterial removal interventions at processing are primarily responsible for the similar levels of contamination of CONV and RWA carcasses with resistant bacteria. These interventions are commonly employed to significantly reduce the overall microbial load, including resistant bacteria carried from the pre-harvest stage [8,46,47,48]. Similar trends are evident in empirical data. Alexander et al. characterized changes in antibiotic-resistant *E. coli* in “farm-to-fork” production of cattle with a known antibiotic administration history [44]. Their results showed that levels of both ampicillin- and tetracycline-resistant *E. coli* were greater in the feces of antibiotic-exposed steer than in the feces of antibiotic-free steers before shipping from the feedlot to the processing plant. However, this difference decreased gradually as the processing chain progressed, and similar levels of resistant *E. coli* were detected in raw ground beef derived from antibiotic-exposed and antibiotic-free animals. Taken together with this empirical evidence, our prediction indicates that antibiotic use at the feedlot level may affect the level of resistant *E. coli* in fecal shedding and that the series of interventions/operations implemented during processing may help dilute the effect.

However, caution is needed when interpreting our results in terms of public health concerns about antibiotic use in agriculture. The transmission of antibiotic resistance from food-producing animals to humans through the food consumption pathway, which was the emphasis of this study, is just one possible exposure route. Other transmission routes may include the environment associated with cattle farming operations, including manure, runoff, dust, and air [49]. The findings of a limited effect of beef consumption on BR-EC transmission are not readily extensible to other transmission scenarios. Few quantitative descriptions of the dissemination of antibiotic resistance from environmental pathways to humans are available due to the complicated interactions among cattle, the environment, and humans, which highlights the clear need for QMEA or risk assessment models to address environmental contributions [50]. In a risk assessment focused on evaluating the dissemination of cattle manure-borne *E. coli* O157:H7 to humans through multiple exposure pathways, Chapman et al. reported that direct contact with cattle during the high-shedding seasons posed the greatest risk of exposure to *E. coli* O157:H7 for human illness, followed by aquatic recreation, consumption of beef meat, consumption of leafy greens, and contact with soil [51]. The analysis by Chapman et al. may shed light on how to tackle the relative contributions of various routes of BR-EC transmission to humans. Considering the multi-route nature of the antibiotic resistance transmission, it may not be unreasonable to assume that the efficacy of antibiotic control in protecting public health is time-dependent; e.g., higher efficiency might be expected after a longer period of application of judicious antibiotic use, as synergistic effects among multiple transmission routes might become stronger over time. However, the inputs accounting for the difference in resistant *E. coli* between the CONV and RWA settings in the present model were primarily estimated based on studies covering relatively small numbers of cycles of cattle, which makes our model inadequate for interpreting the long-term impact of an antibiotic control intervention.

### 3.3. Significant Factors Controlling Human Exposure to BR-EC

**Figure 3:**
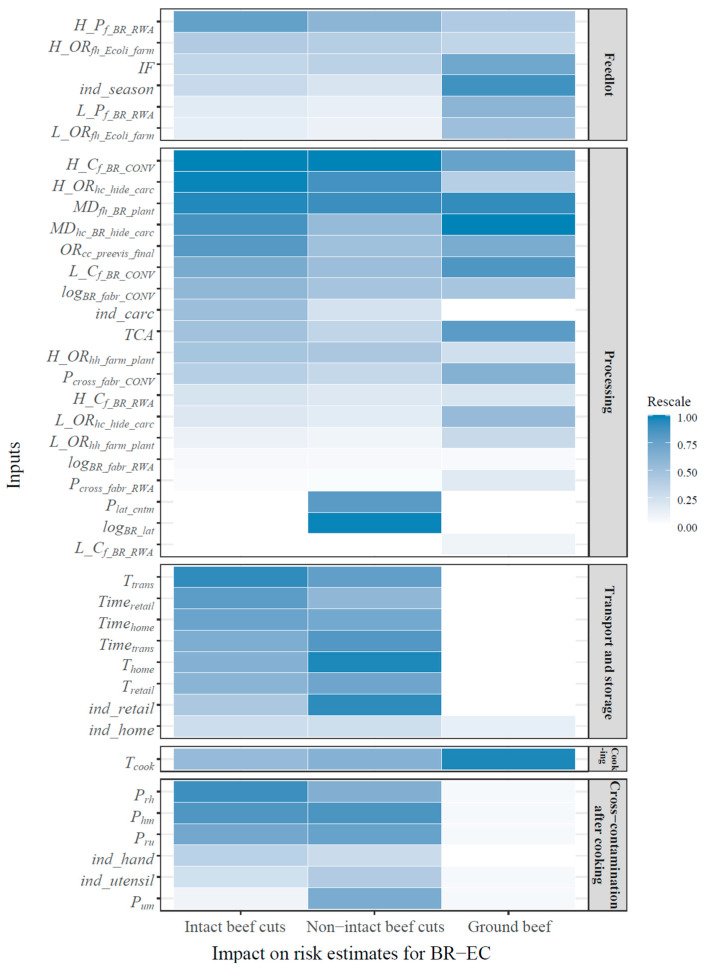
Ranking of the impacts of individual stochastic input variables on the final outputs. The deeper the color, the greater the impact of the input on the output. Refer to Tables S1–S9 for input variable names.

The importance of all stochastic input variables to the model outputs was ranked by measuring and comparing the absolute changes in the output mean by varying individual input values in the sensitivity analysis, as shown in Figure 3, with deeper color indicating greater impact. To better understand the influence of different stages along the supply chain on the microbial load in the end products, all inputs were categorized into five groups corresponding to different modules of the exposure assessment: “feedlot”, “processing”, “transport and storage” (including both retailer-related and consumer-related inputs), “cooking”, and “cross-contamination after cooking”.

In the “feedlot” module, at the top of the list was the probability of high-shedding season (*ind_season*) and the initial bacterial prevalence in RWA or CONV feces (*H/L_P_f_BR_RWA/CONV_*) (Figure 3), followed by the *IF* of BR-EC prevalence from RWA feces to CONV feces and the *OR* quantifying the change in bacterial prevalence from feces to hides at the feedlot (*H/L_OR_fh_Ecoli_farm_*). The importance of season for levels of resistant *E. coli* in cattle feces has been reported previously. Vikram et al. investigated the prevalence and concentration of three types of resistant *E. coli* in beef cattle feces collected from CONV and RWA cattle feedlots in different seasons and concluded that the seasonal effect explained the variations in generic and resistant *E. coli* levels better than the effect of antibiotic use [17]. Specifically, this empirical evidence showed that during summer and fall, i.e., the high-shedding season, the concentration and/or prevalence of resistant *E. coli* was significantly higher than in winter and spring, consistent with the findings of the sensitivity analysis.

Among all modules, the “processing” module accounted for the greatest portion of the supply chain (Tables S2–S6). The most critical variables in this module were the initial microbial loads in RWA or CONV feces (*H/L_C_f_BR_RWA/CONV_*), followed by the variables associated with processing operations, which was reflected by the higher ranks of the *ORs* or *MDs* measuring the contamination changes due to a particular processing step (Figure 3). Commercial interventions currently used at processing plants have been demonstrated to eliminate *E. coli* on beef meat effectively regardless of antibiotic susceptibility [8,48].

As part of secondary processing, variables associated with cross-contamination during fabrication (*P_BR_cross_fabr_RWA/CONV_* and *log_BR_fabr_RWA/CONV_*) played important roles in determining the final levels of exposure to BR-EC via consumption of beef products, particularly ground beef. In addition, tenderization-related inputs (*P_lat_cntm_* and *log_BR_lat_*) were particularly important for non-intact beef cuts. Tenderization may transfer contamination in two different ways, i.e., vertically and laterally. Vertical transfer leads to a microbial redistribution between the surface and interior of a particular meat cut but does not change the presence/absence or total microbial load on/in the cut, whereas lateral transfer can change both prevalence and concentration due to inter-cut cross-contamination [22]. Consequently, only lateral transfer was considered, and no attempt was made to model vertical transfer in the tenderization process. In addition, excluding vertical transfer is likely to have a limited impact on the exposure estimation. After tenderization, the most critical step strongly influencing the exposure estimation is cooking, a major killing step. However, existing thermal inactivation models do not differentiate log reductions based on where microorganisms are located (e.g., internally or externally), as exemplified in relevant risk assessments [16,19,52]. Therefore, the microbial redistribution introduced by vertical transfer would not offer an opportunity for a more accurate prediction of BR-EC. Our predictions showed that tenderization could increase human exposure to BR-EC from consuming non-intact beef cuts compared with intact cuts, indicating that decontamination interventions during tenderization are critically important to control the final number of BR-EC that may be ingested by consumers of non-intact beef cuts.

The “transport and storage” module covered both retailer- and consumer-related input variables. It appears that BR-EC exposure was not sensitive to retailer-related variables (*T_retail_* and *Time_retail_*), as neither of these inputs was deeply colored in the heat map. However, this result does not diminish the importance of retailers to meat safety. U.S. retailers have an excellent record of temperature control. Hence, varying *T_retail_* and *Time_retail_* within their expected range was anticipated to have a limited impact on the mean change in the output.

Consumer-related input variables along the supply chain were covered in three modules (Figure 3): “transport and storage” (home storage), “cooking”, and “cross-contamination after cooking”. Higher-ranked variables were refrigeration storage temperature (*T_home_*), time at home (*Time_home_*), internal cooking temperature (*T_cook_*), proportion of organisms transferred from raw meat to hands/utensils (*P_rh_* and *P_ru_*), and proportion of organisms transferred from contaminated hands/utensils to cooked meat (*P_hm_* and *P_um_*). Cooking temperature (*T_cook_*) was significantly important for the exposure estimates of BR-EC in all beef products.

The impact of cooking temperature on BR-EC in beef meat is of particular interest. Altering *T_cook_* resulted in a variation in the average number of BR-EC of 24.26 CFU/serving of ground beef at the time of ingestion. However, the analysis of various antibiotic use scenarios predicted that the maximum reduction of the mean number of BR-EC ingested through restriction of antibiotic use was 0.69 CFU/serving of ground beef (Figure 2). This result indicates that interventions at other steps along the beef production and preparation continuum, such as appropriate cooking practices, may offer more effective options for consumers to control foodborne exposure to resistant bacteria, consistent with previous risk assessment predictions [23].

The importance of the input variables from the “cross-contamination after cooking” module for BR-EC contamination was greater for beef cuts than for ground beef. In the present model, the same transfer coefficients among raw meat, hands/utensils, and cooked meat meals were applied for various beef products. However, the difference in serving sizes between beef cuts (227 g) and ground beef (85 g) may explain the differences in the importance of cross-contamination-related variables. Overall, the sensitivity analysis suggested that consumers’ cooking/hygiene behaviors in food preparation should be emphasized to reduce BR-EC exposure through the consumption of beef meals.

### 3.4. General Discussion of the Model’s Development

The sensitivity analysis evaluated stochastic variables representing both naturally occurring variability and uncertainty due to lack of information. For most of the input variables at the feedlot and processing stages, the model included sources of variability, such as the initial prevalence and concentrations and various *OR*s and *MD*s that were largely summarized using evidence obtained via a comprehensive literature search. Significance of an uncertainty variable usually indicates an important role of that variable in the model’s accurate prediction capability and the need to fill evidence gaps. In this study, several variables related to cross-contamination were described using a non-informative distribution (Uniform (0,1)) due to a lack of knowledge, including the probability of cross-contamination during fabrication (*P_BR_cross_fabr_RWA/CONV_*) and the probability of lateral cross-contamination during tenderization (*P_lat_cntm_*). The effects of the uncertainties surrounding *P_BR_cross_fabr_RWA/CONV_* and *P_lat_cntm_* on the risk estimates may be relatively large because of the high ranks of these variables, as shown in Figure 3. Therefore, more information is needed to provide accurate descriptions of these input variables to enhance model precision. However, incorporating a non-informative distribution for uncertainty variables should not interfere with achieving our research goal. One main objective of our study was to evaluate the importance of antibiotic use in the beef cattle production system for the level of BR-EC contamination in cooked beef meals, which in essence requires a relative comparison of model outputs under different antibiotic use scenarios. For example, when *P_BR_cross_fabr_RWA_* was changed from 0 to 1, the effect of antibiotic use on the number of BR-EC in ground beef changed slightly from 0.16 to 0.15 CFU/serving. Similar outcomes were obtained when modifying other uncertainty variables, regardless of the type of beef product. Therefore, these uncertainty variables are important from the standpoint of model prediction and are sufficient for our purpose even if a lack of information exists.

Another issue associated with the inputs’ uncertainty is potential overconfidence of the model estimates. One-dimensional Monte Carlo simulation was used in this study without an attempt to separate uncertainty (lack of sufficient knowledge) and variability (inherent heterogeneity). Stochastic input variables were incorporated as probability distributions, which were used to describe either variability (no consideration of parameter uncertainty) or uncertainty (some of which were also incorporated as deterministic values). Omitting the uncertainties for those input variables that were identified to represent variability or simplifying the uncertainty distribution by using deterministic values can lead to overconfident estimates of final model outputs. However, this appears less problematic in this study, as estimating the uncertainty bounds on the risk estimates was beyond the focus of this study [53].

One advantage of this QMEA model is the application of an MA approach to synthesize published evidence collected through a comprehensive literature search for model parameterization in the “feedlot” and “processing” modules. This approach can increase confidence in model input estimation and the extrapolation of modeling-based results to the wider real-world scenario. A comprehensive search of existing evidence can identify and detect information from a set of eligible primary studies. The synthesis of data via MA incorporates the diversity of these values from various populations, study designs, and experimental conditions, thereby decreasing the risk of bias due to sparse data collected from one primary study or narrative review and increasing the representativeness of the observed differences between varying production and processing conditions [54]. All stochastic input variables estimated by the described MA approach are summarized in Table S10 with statistical descriptions of their probabilistic distributions and data sources. The MA outputs can also be visualized as forest plots in Figures S1–S10. 

However, the data gaps and model limitations may not be negligible. The baseline model-estimated outputs were affected by the initial microbial loads of *E. coli* in cattle feces at the processing plant (*H/L_C_f_BR_CONV/RWA_*). Data on the concentration of BR-EC are particularly limited, and only one study was used to estimate concentration-related variables, which highlights the urgent need for more studies reporting enumeration data. Another assumption of this model for reality simplification was a linear association between bacterial transfer and initial contamination loads during the secondary processing steps of beef products (described in Section 2.2.2). Evidence has shown that the change in contamination due to a beef processing step or intervention is determined by a complex interaction among the initial contamination level, bacterial type, contact surface, and contact/treatment time [55]. This simplification increased the uncertainty of the QMEA model, and further research is expected to provide more data about the bacterial transfer or reduction associated with the initial contamination level.

## 4. Conclusions

The model described here provides a mathematical representation of the dynamics of BR-EC contamination in a farm-to-fork continuum to estimate human exposure through the consumption of beef products in the U.S. Both the prevalence and concentration of BR-EC were quantified throughout the whole beef supply chain from feedlot to table. This model can be used as a tool by risk managers to enhance awareness of the potential critical points where antibiotic-resistant *E. coli* can be controlled efficiently to reduce public health risks. Compared with beef cuts, ground beef was shown to pose a higher risk of exposure to BR-EC. Efforts should be made in all sectors along the beef supply chain to decrease the potential for human exposure to resistant bacteria via the consumption of beef meat. However, the results presented here suggest that more promising effects might be expected from interventions at the processing and post-processing stages of beef production than from a standalone restriction of antibiotics in beef production systems.

## Data Availability

All details and data supporting the reported results are provided in the main text and Supplementary Materials.

## Supplementary Materials

The following supporting information can be downloaded at https://www.mdpi.com/article/10.3390/microorganisms10030661/s1. References [56,57,58,59,60,61,62,63,64,65,66,67,68,69,70,71,72,73,74,75,76,77,78,79,80,81,82,83,84,85,86] are cited in the supplementary materials. Figure S1: Forest plot for eligible studies used to fit the impact factor of BR-EC prevalence between RWA and CONV feces (*IF*); Figure S2: Forest plot for eligible studies used to fit the transfer ratio of *E. coli* prevalence from feces to hides at the feedlot in the low-shedding season (*L_OR_fh_Ecoli_farm_*); Figure S3: Forest plot for eligible studies used to fit the transfer ratio of *E. coli* prevalence from feces to hides at the feedlot in the high-shedding season (*H_OR_fh_Ecoli_farm_*); Figure S4: Forest plot for eligible studies used to fit the transfer ratio of *E. coli* prevalence from hides at the feedlot to hides sampled immediately before dehiding in the low-shedding season (*L_OR_hh_farm_plant_*); Figure S5: Forest plot for eligible studies used to fit the transfer ratio of *E. coli* prevalence from hides at the feedlot to hides sampled immediately before dehiding in the high-shedding season (*H_OR_hh_farm_plant_*); Figure S6: Forest plot for eligible studies used to fit the transfer factor of BR-EC concentration from feces to hides at the processing plant (*MD_fh_BR_plant_*); Figure S7: Forest plot for eligible studies used to fit the transfer ratio of *E. coli* prevalence from the hide pre-dehiding to the carcass pre-evisceration in the low-shedding season (*L_OR_hc_hide_carc_*); Figure S8: Forest plot for eligible studies used to fit the transfer ratio of *E. coli* prevalence from the hide pre-dehiding to the carcass pre-evisceration in the high-shedding season (*H_OR_hc_hide_carc_*); Figure S9: Forest plot for eligible studies used to fit the transfer factor of BR-EC concentration from the hide pre-dehiding to the carcass pre-evisceration (*MD_hc_BR_hide_carc_*); Figure S10: Forest plot for eligible studies used to fit the transfer ratio of *E. coli* prevalence due to evisceration (*OR_cc_preevis_final_*). Table S1: Summary of input variables for the “feedlot” module, the distribution/calculation of the input variables and evidence sources; Table S2: Summary of input variables for the cattle compositions, the distribution/calculation of the input variables and evidence sources; Table S3: Summary of input variables for the primary processing of beef carcasses in the “processing” module, the distribution/calculation of the input variables and evidence sources; Table S4: Summary of input variables for the fabrication and trimming of the final carcass in the “processing” module, the distribution/calculation of the input variables and evidence sources; Table S5: Summary of input variables for the production of beef cuts in the “processing” module, the distribution/calculation of the input variables and evidence sources; Table S6: Summary of input variables for the production of ground beef in the “processing” module, the distribution/calculation of the input variables and evidence sources; Table S7: Summary of input variables for the “transport and storage” module, the distribution/calculation of the input variables and evidence sources; Table S8: Summary of input variables for the “cooking” module, the distribution/calculation of the input variables and evidence sources; Table S9: Summary of input variables for the “cross-contamination after cooking” module, the distribution/calculation of the input variables and evidence sources; Table S10: Summary of the estimated parameters of the input variables, log OR and MD, in the quantitative microbial exposure assessment (QMEA) model using the random-effects meta-analysis (MA) approach. Text S1: Fitting the odds ratio (*OR*) and logarithmic mean difference (*MD*) to lognormal and normal distributions based on the results from the meta-analysis (MA); Text S2: Fitting the prevalence of BR-EC in RWA feces at the feedlot (*P_f___BR___RWA_*) to a beta-binomial mixture distribution using a hierarchical model.
