# Supplementary material for: A Farm-to-Fork Quantitative Microbial Exposure Assessment of β-Lactam-Resistant Escherichia coli among U.S. Beef Consumers
Source: Microorganisms. 2022 Mar 19;10(3):661. doi: 10.3390/microorganisms10030661 (PMC8952336; doi:10.3390/microorganisms10030661)
Supplement: Supplementary file 1 [file microorganisms-10-00661-s001.zip › microorganisms-1587910-supplementary.pdf]

Supplementary Materials

For

A Farm-to-Fork Quantitative Microbial Exposure Assessment of β-Lactam-Resistant *Escherichia coli* among U.S. Beef Consumers

Yangjunna Zhang[1], John W. Schmidt[2], Terrance M. Arthur[2], Tommy L. Wheeler[2], Qi Zhang[3], Bing Wang[4,*]

[1] Institute of Food Science and Engineering, Hangzhou Medical College, Hangzhou, Zhejiang, 310013, China; yzhan241@hmc.edu.cn

[2] United States Department of Agriculture, Agricultural Research Service, Roman L. Hruska U.S. Meat Animal Research Center, Clay Center, NE, 68933, USA; john.w.schmidt@usda.gov (J.W.S.), terrance.arthur@usda.gov (T.M.A.), tommy.wheeler@usda.gov (T.L.W.)

[3] Department of Mathematics and Statistics, College of Engineering and Physical Sciences, University of New Hampshire, Durham, NH, 03824, USA; qi.zhang2@unh.edu

[4] Department of Food Science and Technology, College of Agricultural Sciences and Natural Resources, University of Nebraska-Lincoln, Lincoln, NE, 68588, USA; bing.wang@unl.edu

* Correspondence: bing.wang@unl.edu; Tel.: +1 (402) 472-2517

Table S1. Summary of input variables for the "feedlot" module, the distribution/calculation of the input variables and evidence sources.

Variable	Description	Unit	Distribution/calculation (U/V)[a]	Reference
Probability of CONV cattle				
P_{CONV}	Percentage of U.S. cattle administered antimicrobials	%	90.1	[14]
Prevalence in RWA feces at feedlot				
ind_season	Type of sampling season, 1 = high (June to September) shedding period, 0 = low (October to May) shedding period	–	Bernoulli (0.33) (V)	[16]
$H/L\_P_{f\_BR\_RWA}$[b]	Prevalence of BR-EC in RWA feces	%	If *ind_season* = 1, 100 × Beta (0.41, 1.07) (V); otherwise, 100 × Beta (1.29, 2.00) (V)	[56-66]
Prevalence from RWA feces to CONV feces at feedlot				
IF	Impact factor (*OR*) of BR-EC prevalence between RWA and CONV feces	–	Lognorm2 (0.62, 1.02) (V), truncated between -3.61 and 7.07[c]	[56,59,63,65]
$P_{f\_BR\_CONV}$	Prevalence of BR-EC in CONV feces	%	refer to Equation (1), where $P_i = P_{f\_BR\_RWA}$, $OR = IF$	–
Prevalence from feces to hides at feedlot				
$H/L\_OR_{fh\_Ecoli\_farm}$[b]	Transfer ratio of *E. coli* prevalence from feces to hides at feedlot	–	If *ind_season* = 1, Lognorm2 (-0.18, 1.08) (V), truncated between -4.81 and 6.05[c]; otherwise, Lognorm2 (1.59, 1.11) (V), truncated between -6.46 and 4.74[c]	[8,67-72]
$P_{h\_BR\_RWA}$	Prevalence of BR-EC on RWA hides at feedlot	%	refer to Equation (1), where $P_i = P_{f\_BR\_RWA}$, $OR = OR_{fh\_Ecoli\_farm}$	–
$P_{h\_BR\_CONV}$	Prevalence of BR-EC on CONV hides at feedlot	%	refer to Equation (1), where $P_i = P_{f\_BR\_CONV}$, $OR = OR_{fh\_Ecoli\_farm}$	–

[a] U - uncertainty; V - variability.

[b] *H* - high shedding season; *L* - low shedding season.

[c] Lognorm2 (μ, σ) represented the lognormal distribution with specified mean and standard deviation generated from the "logged" values of the distribution. Truncation was conducted by discarding the values exceeding the restricted range and re-allocating the "lost" probability proportionally across the remaining range

between min. and max. For the distributions fitted by MA approach, the upper and lower limits of 95% confidence intervals of observations from empirical studies were selected as the truncation boundaries; otherwise, the observed min. and max. from empirical studies were used as the truncation boundaries.

Table S2. Summary of input variables for the cattle compositions, the distribution/calculation of the input variables and evidence sources.

Variable	Description	Unit	Distribution/calculation (U/V)[a]	Reference
ind_carc	Indicator of carcass type		Discrete ([1,2,3,4],[0.548,0.256,0.18,0.016])[b] (V), where 1 – 4 represent four types of cattle: steer, heifer, dairy, and bull; 0.548 – 0.016 represent their corresponding slaughter proportions annually in the U.S.	[73]
W_{carc}	Chilled carcass weight	kg	If *ind_carc* = 1, Normal (404, 7.90) (V); if *ind_carc* = 2, Normal (373, 7.72) (V); if *ind_carc* = 3, Normal (292, 4.14) (V); otherwise, Normal (409, 7.26) (V). Truncated to min. = 0[c]	[73]
F_{cuts_carc}	Fraction of chilled carcass weight to beef cuts	%	IF *ind_carc* = 1 or 2, 67; if *ind_carc* = 3, 11; otherwise, 0	[16,73-75]
F_{trim_carc}	Fraction of chilled carcass weight to trim	%	IF *ind_carc* = 1 or 2, 18; if *ind_carc* = 3, 65; otherwise, 90	[16,73-75]
W_{cuts_carc}	Weight of beef cuts per chilled carcass	kg	$W_{carc} \times F_{cuts\_carc}$	–
W_{trim_carc}	Weight of trim per chilled carcass	kg	$W_{carc} \times F_{trim\_carc}$	–
TSA	Total outside surface area per carcass	cm^2	IF *ind_carc* = 1 or 2, 32000; if *ind_carc* = 3, 23000; otherwise, 37000	[16]
TCA	Total contaminated surface area per carcass pre-fabrication	cm^2	Uniform (30, *TSA*) (U)	[16]
F_{trim_area}	Proportion of total surface area per carcass to trim	%	75	[16]
F_{cuts_area}	proportion of total surface area per carcass to beef cuts	%	100 - F_{trim_area}	–
N	Number of bins to which an individual carcass contributes	–	5	[76]
$W_{trim\_carc\_bin}$	Weight of trim per chilled carcass to one bin	kg	W_{trim_carc} / N	–
W_{trim_bin}	Weight of trim per bin	kg	907	[16]

$W_{grinding}$	Weight of a grinding load	kg	4,536	[16]
c	Stochastic number of chilled carcasses contribute to one bin	carcasses	Int[d] (W_{trim_bin} / $W_{trim\_carc\_bin}$)	–
b	Number of bins contribute to one grinding load	bins	Int[d] ($W_{grinding}$ / W_{trim_bin})	–

[a] U - uncertainty; V - variability.

[b] Discrete ([*X1*, *X2*, …, *Xn*], [*p1*, *p2*, …, *pn*]): discrete distribution with n possible values (*X*'s) and corresponding probabilities (*p*'s).

[c] Truncation was conducted by discarding the values exceeding the restricted range and re-allocating the "lost" probability proportionally across the remaining range greater than min. The observed min. from empirical studies were used as the truncation boundary.

[d] Int () returns the integer of the calculation.

Table S3. Summary of input variables for the primary processing of beef carcasses in the "processing" module, the distribution/calculation of the input variables and evidence sources.

Variable	Description	Unit	Distribution/calculation (U/V)[a]	Reference
Prevalence from hides at feedlot to hides at the processing plant				
$H/L\_OR_{hh\_farm\_plant}$[b]	Transfer ratio of *E. coli* prevalence from hides at feedlot to hides immediately sampled pre-dehiding	–	If *ind_season* = 1, Lognorm2 (0.60, 1.47) (V), truncated between -4.41 and 8.85[c]; otherwise, Lognorm2 (2.19, 0.73) (V), truncated between -3.95 and 6.46[c]	[8,67-69,77]
$P_{h\_BR\_plant\_RWA}$	Prevalence of BR-EC on RWA hides pre-dehiding	%	refer to Equation (1), where $P_i = P_{h\_BR\_RWA}$, $OR = OR_{hh\_farm\_plant}$	–
$P_{h\_BR\_plant\_CONV}$	Prevalence of BR-EC on CONV hides pre-dehiding	%	refer to Equation (1), where $P_i = P_{h\_BR\_CONV}$, $OR = OR_{hh\_farm\_plant}$	–
Concentration in feces at the processing plant				
$H/L\_C_{f\_BR\_RWA}$[b]	Concentration of BR-EC in RWA feces pre-dehiding	$\log_{10}$ CFU/g	If *ind_season* = 1, Pert (-2, 0.65, 4.37) (V); otherwise, Pert (-2, -2, 0.65) (V)	[17]
$H/L\_C_{f\_BR\_CONV}$[b]	Concentration of BR-EC in CONV feces pre-dehiding	$\log_{10}$ CFU/g	If *ind_season* = 1, Pert (-2, 0.65, 4.97) (V), Pert (-2, -2, 4.55) (V)	[17]
Concentration from feces to hides at the processing plant				
$MD_{fh\_BR\_plant}$	Transfer factor of BR-EC concentration from feces to hides at processing plant	$\log_{10}$ CFU	Normal (0.38, 1.01) (V), truncated between -0.83 and 1.23[e]	[8]
$C_{h\_BR\_plant\_RWA}$	Concentration of BR-EC on RWA hides pre-dehiding	$\log_{10}$ CFU/100cm^2	refer to Equation (2), where $C_i = C_{f\_BR\_RWA}$, $MD = MD_{fh\_BR\_plant}$	–
$C_{h\_BR\_plant\_CONV}$	Concentration of BR-EC on CONV hides pre-dehiding	$\log_{10}$ CFU/100cm^2	refer to Equation (2), where $C_i = C_{f\_BR\_CONV}$, $MD = MD_{fh\_BR\_plant}$	–

Prevalence from hides at the processing plant to carcass pre-evisceration				
$H/L\_OR_{hc\_hide\_carc}$[b]	Transfer ratio of *E. coli* prevalence from hide pre-dehiding to carcass pre-evisceration	–	If *ind_season* = 1, Lognorm2 (-4.35, 3.31) (V), truncated between -13.94 and 3.93[c]; otherwise, Lognorm2 (-3.47, 2.22) (V), truncated between -10.69 and 2.10[c]	[8,67,78,79]
$P_{c\_BR\_preevis\_\_RWA}$	Prevalence of BR-EC on RWA carcass pre-evisceration	%	refer to Equation (1), where $P_i = P_{h\_BR\_plant\_RWA}$, $OR = OR_{hc\_hide\_carc}$	–
$P_{c\_BR\_preevis\_CONV}$	Prevalence of BR-EC on CONV carcass pre-evisceration	%	refer to Equation (1), where $P_i = P_{h\_BR\_plant\_CONV}$, $OR = OR_{hc\_hide\_carc}$	–
Concentration from hides at the processing plant to carcass pre-evisceration				
$MD_{hc\_BR\_hide\_carc}$	Transfer factor of BR-EC concentration from hides pre-dehiding to carcass pre-evisceration	log_{10} CFU	Normal (1.72,1.15) (V), truncated between 0.77 and 2.60[e]	[8]
$C_{c\_BR\_preevis\_RWA}$	Concentration of BR-EC on RWA carcass surface pre-evisceration	log_{10} CFU/100cm^2	refer to Equation (2), where $C_i = C_{h\_BR\_plant\_RWA}$, $MD = MD_{hc\_BR\_hide\_carc}$	–
$C_{c\_BR\_preevis\_CONV}$	Concentration of BR-EC on CONV carcass surface pre-evisceration	log_{10} CFU/100cm^2	refer to Equation (2), where $C_i = C_{h\_BR\_plant\_CONV}$, $MD = MD_{hc\_BR\_hide\_carc}$	–
Prevalence from pre-eviscerated carcass to final carcass				
$OR_{cc\_preevis\_final}$	Transfer ratio of *E. coli* prevalence due to evisceration	–	Lognorm2 (-2.82, 1.86) (V), truncated between -10.70 and 3.93[c]	[8,20]
$P_{c\_BR\_final\_RWA}$	Prevalence of BR-EC on final RWA carcass	%	refer to Equation (1), where $P_i = P_{c\_BR\_preevis\_RWA}$, $OR = OR_{cc\_preevis\_final}$	–
$P_{c\_BR\_final\_CONV}$	Prevalence of BR-EC on final CONV carcass	%	refer to Equation (1), where $P_i = P_{c\_BR\_preevis\_CONV}$, $OR = OR_{cc\_preevis\_final}$	–
Concentration from pre-eviscerated carcass to final carcass				

$MD_{cc\_BR\_preevis\_final}$	Transfer factor of BR-EC concentration due to evisceration	log_{10} CFU	0.66	[8]
$C_{c\_BR\_final\_RWA}$	Concentration of BR-EC on final RWA carcass	log_{10} CFU/100cm^2	refer to Equation (2), where $C_i = C_{c\_BR\_preevis\_RWA}$, $MD = MD_{cc\_BR\_preevis\_final}$	–
$C_{c\_BR\_final\_CONV}$	Concentration of BR-EC on final CONV carcass	log_{10} CFU/100cm^2	refer to Equation (2), where $C_i = C_{c\_BR\_preevis\_CONV}$, $MD = MD_{cc\_BR\_preevis\_final}$	–

[a] U - uncertainty; V - variability.

[b] *H* - high shedding season; *L* - low shedding season.

[c] Lognorm2 (μ, σ) represented the lognormal distribution with specified mean and standard deviation generated from the "logged" values of the distribution. Truncation was conducted by discarding the values exceeding the restricted range and re-allocating the "lost" probability proportionally across the remaining range between min. and max. For the distributions fitted by MA approach, 95% predictive intervals were selected as the truncation boundaries.

[d] This distribution was obtained by fitting empirical data via the @Risk 7.5 distribution fitting tool. The best-fitting distribution was selected based on the Akaike information criterion (AIC) statistics. The observed min. and max. from empirical studies were used as the truncation boundaries.

[e] For the distributions fitted by MA approach, the upper and lower limits of 95% confidence intervals of observations from empirical studies were selected as the truncation boundaries.

Table S4. Summary of input variables for the fabrication and trimming of the final carcass in the "processing" module, the distribution/calculation of the input variables and evidence sources.

Variable	Description	Unit	Distribution/calculation (U/V)[a]	Reference
Prevalence change during fabrication and trimming				
$P_{BR\_cross\_fabr\_RWA/CONV}$	Probability of cross-contamination of BR-EC to a particular carcass	%	Uniform (0, 100) (U)	–
Concentration change during fabrication and trimming				
log_{BR_fabr}	Log increase of BR-EC due to cross-contamination during fabrication	log_{10} CFU	If *ind_season* = 1, Pert (0, 0.22, 1.5) (V); otherwise, Pert (0, 0.33, 1.5) (V)	[16]
$C_{c\_BR\_postfabr\_RWA}$	Concentration of BR-EC on a RWA carcass post-fabrication	CFU/100cm[2]	$P_{c\_BR\_final\_RWA} \times (1 - P_{BR\_cross\_fabr\_RWA/CONV} \times 10^{C_{c\_BR\_final\_RWA}} + P_{c\_BR\_final\_RWA} \times P_{BR\_cross\_fabr\_RWA/CONV} \times 10^{(C_{c\_BR\_final\_RWA}+logBR\_fabr)} + (1 - P_{c\_BR\_final\_RWA}) \times (1 - P_{BR\_cross\_fabr\_RWA/CONV}) \times 0 + (1 - P_{c\_BR\_final\_RWA}) \times P_{BR\_cross\_fabr\_RWA/CONV} \times 10^{(-100 + logBR\_fabr)}$	–
$C_{c\_BR\_postfabr\_CONV}$	Concentration of BR-EC on a CONV carcass post-fabrication	CFU/100cm[2]	$P_{c\_BR\_final\_CONV} \times (1 - P_{BR\_cross\_fabr\_RWA/CONV}) \times 10^{C_{c\_BR\_final\_CONV}} + P_{c\_BR\_final\_CONV} \times P_{BR\_cross\_fabr\_RWA/CONV} \times 10^{(C_{c\_BR\_final\_CONV}+logBR\_fabr)} + (1 - P_{c\_BR\_final\_CONV}) \times (1 - P_{BR\_cross\_fabr\_RWA/CONV}) \times 0 + (1 - P_{c\_BR\_final\_CONV}) \times P_{BR\_cross\_fabr\_RWA/CONV} \times 10^{(-100 + logBR\_fabr)}$	–
Concentration on the outside surface after fabrication and trimming				
$C_{c\_BR\_postfabr}$	Concentration of BR-EC on a non-specific carcass post-fabrication	CFU/100cm[2]	$P_{CONV} \times C_{c\_BR\_postfabr\_CONV} + (1 - P_{CONV}) \times C_{c\_BR\_postfabr\_RWA}$	–

[a] U - uncertainty; V - variability.

Table S5. Summary of input variables for the production of beef cuts in the "processing" module, the distribution/calculation of the input variables and evidence sources.

Variable	Description	Unit	Distribution/calculation (U/V)[a]	Reference
Processing of intact beef cuts				
A_{cuts_carc}	Total surface area per carcass to beef cuts	cm^2	$TSA \times F_{cuts\_area}$	–
TCA_{cuts}	Contaminated surface area of beef cuts per carcass	cm^2	$(TCA/TSA) \times A_{cuts\_carc}$	–
N_{BR_int}	Number of BR-EC on intact beef cuts	CFU	Poisson ($TCA_{cuts} \times C_{c\_BR\_postfabr}$ /100)	–
$C_{N\_BR\_int}$	Concentration of BR-EC on intact beef cuts per gram	CFU/g	$\frac{N_{BR\_int}}{W_{cuts\_carc} \times 1000}$	–
C_{BR_int}	Concentration of BR-EC on intact beef cuts	log_{10} CFU/g	If $C_{N\_BR\_int} > 0$, $log_{10}(C_{N\_BR\_int})$; otherwise, -100[b]	–
Processing of non-intact beef cuts (tenderization)				
P_{lat_cntm}	Probability of lateral cross-contamination during tenderization	%	Uniform (0, 100) (U)	–
$ind\_P_{lat\_cmtm}$	Indicator of occurrence of lateral cross-contamination of BR-EC, 1 = occur, 0 = not occur	–	Bernoulli (P_{lat_cntm})	–
log_{BR_lat}	Log change of BR-EC on non-intact beef cuts due to tenderization	log_{10} CFU	Uniform (0, 1.5) (U)	[22]
C_{BR_nonint}	Concentration of BR-EC on non-intact beef cuts	log_{10} CFU/g	IF C_{BR_int} = -100 and $ind\_P_{lat\_cmtm}$ = 1, -100 + log_{BR_lat}; IF C_{BR_int} = -100 and $ind\_P_{lat\_cmtm}$ = 0, -100[a]; IF $C_{BR\_int} \neq$ -100 and $ind\_P_{lat\_cmtm}$	–

	= 1, C_{BR_int} + log_{BR_lat}; IF $C_{BR\_int} \neq -100$ and $ind\_P_{lat\_cmtm}$ = 0, C_{BR_int} - log_{BR_lat}

[a] U - uncertainty; V - variability.

[b] If $C_{N\_BR\_int} = 0$, $\log_{10}(C_{N\_BR\_int})$ will return -∞; To avoid error message, -100 was used to replace -∞ at this situation.

Table S6. Summary of input variables for the production of ground beef in the "processing" module, the distribution/calculation of the input variables and evidence sources.

Variable	Description	Unit	Distribution/calculation	Reference
A_{trim_carc}	Total surface area per carcass to trim	cm^2	$TSA \times F_{trim\_area}$	–
AW_{trim}	Total surface area per kg per carcass to trim	cm^2/kg	$A_{trim\_carc} / W_{trim\_carc}$	–
$A_{trim\_carc\_bin}$	Total surface area of trim per carcass to one bin	cm^2	$AW_{trim} \times W_{trim\_carc\_bin}$	–
TCA_{trim_bin}	Contaminated surface area of trim per carcass to one bin	cm^2	$(TCA/TSA) \times A_{trim\_carc\_bin}$	–
$N_{BR\_trim\_carc\_bin}$	Number of BR-EC in trim from carcass to one bin	CFU	Poisson ($TCA_{trim\_bin} \times C_{c\_BR\_postfabr}$ /100)	–
N_{BR_bin}	Number of BR-EC in trim per bin	CFU	$\sum_0^c N_{BR\_trim\_carc\_bin}$	–
N_{BR_load}	Number of BR-EC in one grinding load	CFU	$\sum_1^b N_{BR\_bin}$	–
C_{BR_gb}	Concentration of BR-EC in ground beef	$\log_{10}$ CFU/g	If $N_{BR\_load} > 0$, $\log_{10}(\frac{N_{BR\_load}}{W_{grinding} \times 1000})$; otherwise, -100[a]	–

[a] If $N_{BR\_load} = 0$, $\log_{10}(N_{BR\_load} / (W_{grinding} \times 1000))$ will return -∞; To avoid error message, -100 was used to replace -∞ at this situation.

Table S7. Summary of input variables for the "transport and storage" module, the distribution/calculation of the input variables and evidence sources.

Variable	Description	Unit	Distribution/calculation (U/V)[a]	Reference
At retail				
ind_retail	Indicator of retail storage type, 1 = fridge, 0 = freezer	–	Bernoulli (0.83)	[19,28]
T_{retail}	Fridge storage temperature	°C	Laplace (3.33, 2.66) (V), truncated between 0 and 19.4[b]	[28]
$Time_{retail}$	Retail storage time on display	hour	24 × Exponential (Uniform (0.5, 1.5)) (V), truncated between 0 and 24 × 14[b]	[16,29]
T_{obs}	Minimum temperature allowing prediction of *E. coli* growth in/on beef	°C	10.08	[24]
$y1_{max}$	Maximum population density during fridge storage at retail	log_{10} CFU/g	$9.41 + (-1.23 \times 10^{-5} \times T_{retail}^3)$	[24]
At retail – contamination of intact and non-intact beef cuts				
$r1_{max}$	Maximum specific growth rate of *E. coli* in beef cuts during fridge storage at retail	log_{10} CFU/hour	refer to Equation (3), where $T = T_{retail}$	[80]
$\lambda1_{max}$	Lag phase duration of *E. coli* in beef cuts during fridge storage at retail	hour	If $T_{retail} \geq T_{obs}$, refer to Equation (4), where $T = T_{retail}$; otherwise, 0[c]	[80]
$F1(t)$	Intermediate factor for beef cuts during fridge storage at retail	–	$Time_{retail} + \frac{1}{r1_{max}} \ln\left(e^{-r1_{max} \times Time_{retail}} + e^{-r1_{max} \times \lambda1_{max}} - e^{-r1_{max} \times Time_{retail} - r1_{max} \times \lambda1_{max}}\right)$	[25]
$log_{BR\_int\_retail}$	Maximum increase of BR-EC during fridge storage of intact beef cuts at retail	log_{10} CFU/g	If $C_{BR\_int} \neq -100$ & $\lambda1_{max} > Time_{retail}$, $r1_{max} \times F1(t) - \ln\left(1 + \frac{e^{r1_{max} F1(t)} - 1}{e^{y1_{max} - C_{BR\_int}}}\right)$; otherwise, 0	[25]

$C_{BR\_int\_retail}$	Concentration of BR-EC on intact beef cuts after fridge storage at retail	log_{10} CFU/g	$C_{BR\_int} + log_{BR\_int\_retail}$	–
$log_{BR\_nonint\_retail}$	Maximum increase of BR-EC during fridge storage of non-intact beef cuts at retail	log_{10} CFU/g	If $C_{BR\_nonint} \neq -100$ & $\lambda 1_{max} > Time_{retail}$, $r1_{max} \times F1(t) - \ln\left(1 + \frac{e^{r1_{max}F1(t)}-1}{e^{y1_{max}-C_{BR\_nonint}}}\right)$; otherwise, 0	[25]
$C_{BR\_nonint\_retail}$	Concentration of BR-EC on non-intact beef cuts after fridge storage at retail	log_{10} CFU/g	$C_{BR\_nonint} + log_{BR\_nonint\_retail}$	–
At retail – contamination of ground beef				
$r2_{max}$	Maximum specific growth rate of *E. coli* in ground beef during fridge storage at retail	log_{10} CFU/hour	refer to Equation (5), where $T = T_{retail}$	[24]
$\lambda 2_{max}$	Lag phase duration of *E. coli* in ground beef during fridge storage at retail	hour	If $T_{retail} \geq T_{obs}$, refer to Equation (6), where $T = T_{retail}$; otherwise, 0[c]	[24]
$F2(t)$	Intermediate factor for ground beef during fridge storage at retail	–	$Time_{retail} + \frac{1}{r2_{max}}\ln\left(e^{-r2_{max}\times Time_{retail}} + e^{-r2_{max}\times\lambda 2_{max}} - e^{-r2_{max}\times Time_{retail} - r2_{max}\times\lambda 2_{max}}\right)$	[25]
$log_{BR\_gb\_retail}$	Maximum increase of BR-EC during fridge storage of ground beef at retail	log_{10} CFU/g	If $C_{BR\_gb} \neq -100$ & $\lambda 2_{max} > Time_{retail}$, $r2_{max} \times F2(t) - \ln\left(1 + \frac{e^{r2_{max}F2(t)}-1}{e^{y1_{max}-C_{BR\_gb}}}\right)$; otherwise, 0	[25]
$C_{BR\_gb\_retail}$	Concentration of BR-EC in ground beef after fridge storage at retail	log_{10} CFU/g	$C_{BR\_gb} + log_{BR\_gb\_retail}$	–
Transport from retail to home				
T_{trans}	Transport temperature ≥ 0 °C from retail to home	°C	Loglogistic (-22.96, 29.42, 16.77) (V), truncated between 0 and 20[b]	[28]

$Time_{trans}$	Transport time from retail to home	hour	Lognormal (1.33, 0.51, Shift (-0.13)) (V), truncated between 0.43 and 3.83[b]	[28]
$y2_{max}$	Maximum population density during transport from retail to home	log_{10} CFU/g	$9.41 + (-1.23 \times 10^{-5} \times T_{trans}^3)$	[24]
Transport from retail to home – contamination of intact and non-intact beef cuts				
$r3_{max}$	Maximum specific growth rate of *E. coli* in beef cuts during transport from retail to home at T_{trans}	log_{10} CFU/hour	refer to Equation (3), where $T = T_{trans}$	[80]
$\lambda 3_{max}$	Lag phase duration of *E. coli* in beef cuts during transport from retail to home at T_{trans}	hour	If $T_{trans} \geq T_{obs}$, refer to Equation (4), where $T = T_{trans}$; otherwise, 0[c]	[80]
$F3(t)$	Intermediate factor for beef cuts during transport from retail to home at T_{trans}	–	$Time_{trans} + \frac{1}{r3_{max}} \ln(e^{-r3_{max} \times Time_{trans}} + e^{-r3_{max} \times \lambda 3_{max}} - e^{-r3_{max} \times Time_{trans} - r3_{max} \times \lambda 3_{max}})$	[25]
$log_{BR\_int\_trans}$	Maximum increase of BR-EC during transport of intact beef cuts from retail to home	log_{10} CFU/g	If $C_{BR\_int\_retail} \neq -100$ & $\lambda 3_{max} > Time_{trans}$, $r3_{max} \times F3(t) - \ln\left(1 + \frac{e^{r3_{max} F3(t)} - 1}{e^{y2_{max} - C_{BR\_int\_retail}}}\right)$; otherwise, 0	[25]
$C_{BR\_int\_trans}$	Concentration of BR-EC on intact beef cuts after transport	log_{10} CFU/g	$C_{BR\_int\_retail} + log_{BR\_int\_trans}$	–
$log_{BR\_nonint\_trans}$	Maximum increase of BR-EC during transport of non-intact beef cuts from retail to home	log_{10} CFU/g	If $C_{BR\_nonint\_retail} \neq -100$ & $\lambda 3_{max} > Time_{trans}$, $r3_{max} \times F3(t) - \ln\left(1 + \frac{e^{r3_{max} F3(t)} - 1}{e^{y2_{max} - C_{BR\_nonint\_retail}}}\right)$; otherwise, 0	[25]
$C_{BR\_nonint\_trans}$	Concentration of BR-EC on non-intact beef cuts after transport	log_{10} CFU/g	$C_{BR\_nonint\_retail} + log_{BR\_nonint\_trans}$	–
Transport from retail to home – contamination of ground beef				

$r4_{max}$	Maximum specific growth rate of *E. coli* in ground beef during transport from retail to home at T_{trans}	$\log_{10}$ CFU/hour	refer to Equation (5), where $T = T_{trans}$	[24]
$\lambda 4_{max}$	Lag phase duration of *E. coli* in ground beef during transport from retail to home at T_{trans}	hour	If $T_{trans} \geq T_{obs}$, refer to Equation (6), where $T = T_{trans}$; otherwise, 0[c]	[24]
$F4(t)$	Intermediate factor for ground beef during transport from retail to home at T_{trans}	–	$Time_{trans} + \frac{1}{r4_{max}}\ln\left(e^{-r4_{max}\times Time_{trans}} + e^{-r4_{max}\times\lambda4_{max}} - e^{-r4_{max}\times Time_{trans} - r4_{max}\times\lambda4_{max}}\right)$	[25]
$log_{BR\_gb\_trans}$	Maximum increase of BR-EC during transport of ground beef from retail to home	$\log_{10}$ CFU/g	If $C_{BR\_gb\_retail} \neq -100$ & $\lambda4_{max} > Time_{trans}$, $r4_{max} \times F4(t) - \ln\left(1 + \frac{e^{r4_{max}F4(t)} - 1}{e^{y2_{max} - C_{BR\_gb\_retail}}}\right)$; otherwise, 0	[25]
$C_{BR\_gb\_trans}$	Concentration of BR-EC in ground beef after transport	$\log_{10}$ CFU/g	$C_{BR\_gb\_retail} + log_{BR\_gb\_trans}$	–
At home				
T_{home}	Fridge storage temperature at home	°C	Cumulative (-3.33, 18.33, [0, 1.67, 3.33, 5, 6.67, 8.33, 10, 11.67, 13.33, 15], [0.105, 0.235, 0.515, 0.835, 0.935, 0.975, 0.995, 0.997, 0.999, 1] (V)[e]	[28]
$y3_{max}$	Maximum population density during home storage	$\log_{10}$ CFU/g	$9.41 + (-1.23 \times 10^{-5} \times T_{home}^3)$	[24]
At home – contamination of intact and non-intact beef cuts				
ind_home	Indicator of storage type of beef cuts, 1 = fridge, 0 = freezer	–	Bernoulli (0.16)	[30]
$Time_{home}$	Fridge storage time of beef cuts at home	hour	Cumulative (0, 336, [24, 72, 168, 336], [0.416, 0.851, 0.941, 1]) (V)[e]	[30]

$r5_{max}$	Maximum specific growth rate of *E. coli* in beef cuts during fridge storage at home	$\log_{10}$ CFU/hour	refer to Equation (3), where $T = T_{home}$	[80]
$\lambda5_{max}$	Lag phase duration of *E. coli* in beef cuts during fridge storage at home	hour	If $T_{home} \geq T_{obs}$, refer to Equation (4), where $T = T_{home}$; otherwise, 0[c]	[80]
$F5(t)$	Intermediate factor for beef cuts during fridge storage at home	–	$Time_{home} + \frac{1}{r5_{max}} \ln\left(e^{-r5_{max} \times Time_{home}} + e^{-r5_{max} \times \lambda5_{max}} - e^{-r5_{max} \times Time_{home} - r5_{max} \times \lambda5_{max}}\right)$	[25]
$log_{BR\_int\_home}$	Maximum increase of BR-EC during fridge storage of intact beef cuts at home	$\log_{10}$ CFU/g	If $C_{BR\_int\_trans} \neq -100$ & $\lambda5_{max} > Time_{home}$, $r5_{max} \times F5(t) - \ln\left(1 + \frac{e^{r5_{max} F5(t)} - 1}{e^{y3_{max} - C_{BR\_int\_trans}}}\right)$; otherwise, 0	[25]
$C_{BR\_int\_home}$	Concentration of BR-EC on intact beef cuts after fridge storage at home	$\log_{10}$ CFU/g	$C_{BR\_int\_trans} + log_{BR\_int\_home}$	–
$log_{BR\_nonint\_home}$	Maximum increase of BR-EC during fridge storage of non-intact beef cuts at home	$\log_{10}$ CFU/g	If $C_{BR\_nonint\_trans} \neq -100$ & $\lambda5_{max} > Time_{home}$, $r5_{max} \times F5(t) - \ln\left(1 + \frac{e^{r5_{max} F5(t)} - 1}{e^{y3_{max} - C_{BR\_nonint\_trans}}}\right)$; otherwise, 0	[25]
$C_{BR\_nonint\_home}$	Concentration of BR-EC on non-intact beef cuts after fridge storage at home	$\log_{10}$ CFU/g	$C_{BR\_nonint\_trans} + log_{BR\_nonint\_home}$	–
At home – contamination of ground beef				
ind_gb_home	Indicator of storage type of ground beef, 1 = fridge, 0 = freezer	–	Bernoulli (0.11)	[30]
$Time_{gb\_home}$	Fridge storage time of ground beef at home	hour	Cumulative (0, 168,[24, 72, 168], [0.38, 0.85, 1] (V)[e]	[30]

$r6_{max}$	Maximum specific growth rate of *E. coli* in ground beef during fridge storage at home	log_{10} CFU/hour	refer to Equation (5), where $T = T_{home}$	[24]
$\lambda 6_{max}$	Lag phase duration of *E. coli* in ground beef during fridge storage at home	hour	If $T_{home} \geq T_{obs}$, refer to Equation (6), where $T = T_{home}$; otherwise, 0[c]	[24]
$F6(t)$	Intermediate factor for ground beef during fridge storage at home	–	$Time_{gb\_home} + \frac{1}{r6_{max}} \ln\left(e^{-r6_{max} \times Time_{gb\_home}} + e^{-r6_{max} \times \lambda 6_{max}} - e^{-r6_{max} \times Time_{gb\_home} - r6_{max} \times \lambda 6_{max}}\right)$	[25]
$log_{BR\_gb\_home}$	Maximum increase of BR-EC during home storage of ground beef	log_{10} CFU/g	If $C_{BR\_gb\_trans} \neq -100$ & $\lambda 6_{max} > Time_{home}$, $r6_{max} \times F6(t) - \ln\left(1 + \frac{e^{r6_{max} F6(t)} - 1}{e^{y3_{max} - C_{BR\_gb\_trans}}}\right)$; otherwise, 0	[25]
$C_{BR\_gb\_home}$	Concentration of BR-EC in ground beef after home storage	log_{10} CFU/g	$C_{BR\_gb\_trans} + log_{BR\_gb\_home}$	–

[a] U - uncertainty; V - variability.

[b] This distribution was obtained by fitting empirical data via the @Risk 7.5 distribution fitting tool. The best-fitting distribution was selected based on the Akaike information criterion (AIC) statistics. Truncation was conducted by discarding the values exceeding the restricted range and re-allocating the "lost" probability proportionally across the remaining range between min. and max. The observed min. and max. from empirical studies were used as the truncation boundaries.

[c] Lag phase duration was set to 0 at temperature lower than T_{obs}.

[d] The observed max. from empirical studies were used as the truncation boundary.

[e] Cumulative (minimum, maximum, [*X1*, *X2*, …, *Xn*],[*p1*, *p2*, …, *pn*]: cumulative distribution with n points between minimum and maximum, with cumulative ascending probability *p* for each *X* value.

Table S8. Summary of input variables for the "cooking" module, the distribution/calculation of the input variables and evidence sources.

Variable	Description	Unit	Distribution/calculation (U/V)[a]	Reference
T_{cook}	Internal temperature of beef cuts during cooking	°C	Normal (69.3, 13.7) (V), truncated between 27 and 138[b]	[28]
Cooking – contamination of intact beef cuts				
$K0_{int}$	Regression coefficient – intact beef cuts	log_{10} CFU/g	-1.24	[19]
$K1_{int}$	Regression coefficient – intact beef cuts	log_{10} CFU/g °C	0.09	[19]
log_{int_cook}	log reduction of *E. coli* on intact beef cuts	log_{10} CFU/g	$K0_{int} + K1_{int} \times T_{cook}$	[19]
$C_{BR\_int\_cook}$	Concentration of BR-EC on intact beef cuts after cooking	CFU/g	If $C_{BR\_int\_home}$ = -100, 0; otherwise, $10^{C_{BR\_int\_home} - log_{int\_cook}}$	–
Cooking – contamination of non-intact beef cuts				
$K0_{nonint}$	Regression coefficient – non-intact beef cuts	log_{10} CFU/g	-1.52	[19]
$K1_{nonint}$	Regression coefficient – non-intact beef cuts	log_{10} CFU/g °C	0.091	[19]
log_{nonint_cook}	log reduction of *E. coli* on non-intact beef cuts	log_{10} CFU/g	$K0_{nonint} + K1_{nonint} \times T_{cook}$	[19]
$C_{BR\_nonint\_cook}$	Concentration of BR-EC on non-intact beef cuts after cooking	CFU/g	If $C_{BR\_nonint\_home}$ = -100, 0; otherwise, $10^{C_{BR\_nonint\_home} - log_{nonint\_cook}}$	–
Cooking – contamination of ground beef				
T_{gb_cook}	Internal temperature of ground beef during cooking	°C	Weibull (7.03, 78.1, Shift(-3.07)) (V), truncated between 26.07 and 102.07[b]	[19,28]

$K0_{gb}$	Regression coefficient – ground beef	log_{10} CFU/g	-10.2	[19]
$K1_{gb}$	Regression coefficient – ground beef	log_{10} CFU/g °C	0.21	[19]
log_{gb_cook}	log reduction of *E. coli* in ground beef	log_{10} CFU/g	$K0_{gb} + K1_{gb} \times T_{gb\_cook}$	[19]
$C_{BR\_gb\_cook}$	Concentration of BR-EC in ground beef after cooking	CFU/g	If $C_{BR\_gb\_home}$ = -100, 0; otherwise, $10^{C_{BR\_gb\_home} - log_{gb\_cook}}$	–

[a] U - uncertainty; V - variability.

[b] Truncation was conducted by discarding the values exceeding the restricted range and re-allocating the "lost" probability proportionally across the remaining range between min. and max. The observed min. and max. from empirical studies were used as the truncation boundaries.

Table S9. Summary of input variables for the "cross-contamination after cooking" module, the distribution/calculation of the input variables and evidence sources.

Variable	Description	Unit	Distribution/calculation (U/V)[a]	Reference
W_{bc}	Recommended beef cuts serving size	g	227	[32]
W_{gb}	Recommended ground beef serving size	g	85	[33]
Cross-contamination from raw meat to hands				
P_{rh}	Proportion of bacteria transferred from raw meat to hands	proportion	Pert (0.011, 0.065, 0.261) (V)	[31]
$C_{BR\_int\_rh}$	Number of BR-EC transferred from raw intact beef cuts to hands	CFU/g	If $C_{BR\_int\_home} \neq$ -100, $P_{rh} \times 10^{C_{BR\_int\_home}}$; otherwise, 0	–
$C_{BR\_nonint\_rh}$	Number of BR-EC transferred from raw non-intact beef cuts to hands	CFU/g	If $C_{BR\_nonint\_home} \neq$ -100, $P_{rh} \times 10^{C_{BR\_nonint\_home}}$; otherwise, 0	–
$C_{BR\_gb\_rh}$	Number of BR-EC transferred from raw ground beef to hands	CFU/g	If $C_{BR\_gb\_home} \neq$ -100, $P_{rh} \times 10^{C_{BR\_gb\_home}}$; otherwise, 0	–
Cross-contamination from raw meat to utensil				
P_{ru}	Proportion of bacteria transferred from raw meat to kitchen utensil	proportion	Pert (0.03, 0.075, 0.309) (V)	[31]
$C_{BR\_int\_ru}$	Number of BR-EC transferred from raw intact beef cuts to kitchen utensil	CFU/g	If $C_{BR\_int\_home} \neq$ -100, $P_{rc} \times 10^{C_{BR\_int\_home}}$; otherwise, 0	–
$C_{BR\_nonint\_ru}$	Number of BR-EC transferred from raw non-intact beef cuts to kitchen utensil	CFU/g	If $C_{BR\_nonint\_home} \neq$ -100, $P_{rc} \times 10^{C_{BR\_nonint\_home}}$; otherwise, 0	–

$C_{BR\_gb\_ru}$	Number of BR-EC transferred from raw ground beef to kitchen utensil	CFU/g	If $C_{BR\_gb\_home} \neq -100$, $P_{rc} \times 10^{C_{BR\_gb\_home}}$; otherwise, 0	–
Cross-contamination from contaminated hands to cooked meat				
ind_hand	Indicator of cleaning hands after handling raw meat, 1 = wash, 0 = not wash	–	Bernoulli (0.38)	[81]
P_{hm}	Proportion of bacteria transferred from contaminated hands to cooked meat	proportion	Pert (0.001, 0.089, 0.529) (V)	[31]
$C_{BR\_int\_hand}$	Number of BR-EC transferred from contaminated hands to cooked intact beef cuts	CFU/g	If *ind_hand* = 1, 0; otherwise, $C_{BR\_int\_rh} \times P_{hm}$	–
$C_{BR\_nonint\_hand}$	Number of BR-EC transferred from contaminated hands to cooked non-intact beef cuts	CFU/g	If *ind_hand* = 1, 0; otherwise, $C_{BR\_nonint\_rh} \times P_{hm}$	–
$C_{BR\_gb\_hand}$	Number of BR-EC transferred from contaminated hands to cooked ground beef	CFU/g	If *ind_hand* = 1, 0; otherwise, $C_{BR\_gb\_rh} \times P_{hm}$	–
Cross-contamination from contaminated utensil to cooked meat				
ind_utensil	Indicator of cleaning kitchen utensil after treating raw meat, 1 = not clean, 0 = clean	–	Bernoulli (0.42)	[81]
P_{um}	Proportion of bacteria transferred from contaminated utensil to cooked meat	proportion	Pert (0.105, 0.194, 0.424) (V)	[31]
$C_{BR\_int\_utensil}$	Number of BR-EC transferred from contaminated utensil to cooked intact beef cuts	CFU/g	If *ind_utensil* = 0, 0; otherwise, $C_{BR\_int\_ru} \times P_{um}$	–

$C_{BR\_nonint\_utensil}$	Number of BR-EC transferred from contaminated utensil to cooked non-intact beef cuts	CFU/g	If *ind_utensil* = 0, 0; otherwise, $C_{BR\_nonint\_ru} \times P_{um}$	–
$C_{BR\_gb\_utensil}$	Number of BR-EC transferred from contaminated utensil to cooked ground beef	CFU/g	If *ind_utensil* = 0, 0; otherwise, $C_{BR\_gb\_ru} \times P_{um}$	–
Risk estimates				
N_{BR_int}	Final number of BR-EC on cooked intact beef cuts	CFU/serving	$(C_{BR\_int\_cook} + C_{BR\_int\_hand} + C_{BR\_int\_utensil}) \times W_{bc}$	–
N_{BR_nonint}	Final number of BR-EC on cooked non-intact beef cuts	CFU/serving	$(C_{BR\_nonint\_cook} + C_{BR\_nonint\_hand} + C_{BR\_nonint\_utensil}) \times W_{bc}$	–
N_{BR_gb}	Final number of BR-EC in cooked ground beef	CFU/serving	$(C_{BR\_gb\_cook} + C_{BR\_gb\_hand} + C_{BR\_gb\_utensil}) \times W_{gb}$	–

[a] U - uncertainty; V - variability.

Table S10. Summary of the estimated parameters of the input variables, log *OR* and *MD*, in the quantitative microbial exposure assessment (QMEA) model, using the random-effects meta-analysis (MA) approach

Variable	MA outputs for estimating QMEA model input variable distributions			Truncation boundary			Distribution	Reference
	Mean of the pooled effect size (μ)	Within-study standard error (se)	Between-study variance (τ^2)	Observed min. and max. effect sizes in MA studies[a]	95% prediction interval of MA estimates[b]	Range of 95% confidence interval across MA studies (Used as the truncation boundaries)[c]		
log OR								
IF - Impact factor of BR-EC prevalence between RWA and CONV feces	0.62	0.19	1.0009	-1.34, 4.26	-1.37, 2.62	-3.61, 7.07	Lognorml2[d] (0.62, 1.02), truncated between -3.61 and 7.07	[56-66]
$L\_OR_{fh\_Ecoli\_farm}$ - Transfer ratio of *E. coli* prevalence from feces to hides at feedlot in low shedding season	1.59	0.40	1.0799	-3.52, 3.48	-0.59, 3.77	-6.46, 4.74	Lognorml2[d] (1.59, 1.11), truncated between -6.46 and 4.74	[8,67-72]
$H\_OR_{fh\_Ecoli\_farm}$ - Transfer ratio of *E. coli* prevalence from feces to hides at feedlot in high shedding season	-0.18	0.28	1.0868	-2.13, 3.19	-2.29, 1.94	-4.81, 6.05	Lognorml2[d] (-0.18, 1.08), truncated between -4.81 and 6.05	
$L\_OR_{hh\_farm\_plant}$ - Transfer ratio of *E. coli* prevalence from hides at feedlot to hides immediately sampled pre-	2.19	0.45	0.3337	0, 3.52	0.76, 3.63	-3.95, 6.46	Lognorml2[d] (2.19, 0.73), truncated between -3.95 and 6.46	[8,67-69,77]

dehiding in low shedding season								
$H\_OR_{hh\_farm\_plant}$ - Transfer ratio of *E. coli* prevalence from hides at feedlot to hides immediately sampled pre-dehiding in high shedding season	0.60	0.48	1.9453	-1.15, 6.00	-2.29, 3.50	-4.41, 8.85	Lognorml2[d] (0.60, 1.47), truncated between -4.41 and 8.85	
$L\_OR_{hc\_hide\_carc}$ - Transfer ratio of *E. coli* prevalence from hide pre-dehiding to carcass pre-evisceration in low shedding season	-3.47	0.77	4.3549	-7.45, -1.13	-7.83, 0.89	-10.69, 2.10	Lognorml2[d] (-3.47, 2.22), truncated between -10.69 and 2.10	[8,67,69,78,82]
$H\_OR_{hc\_hide\_carc}$ - Transfer ratio of *E. coli* prevalence from hide pre-dehiding to carcass pre-evisceration in high shedding season	-4.35	1.04	9.8968	-10.01, 0	-10.85, 2.14	-13.94, 3.93	Lognorml2[d] (-4.35, 3.31), truncated between -13.94 and 3.93	
$OR_{cc\_preevis\_final}$ - Transfer ratio of *E. coli* prevalence due to evisceration	-2.82	0.69	2.9961	-7.76, 0	-6.47, 0.83	-10.70, 3.93	Lognorml2[d] (-2.82, 1.86), truncated between -10.70 and 3.93	[8,20]
MD (log_{10} CFU)								
$MD_{fh\_BR\_plant}$ - Transfer factor of BR-EC concentration	0.38	0.49	0.695	-0.59, 0.89	-1.51, 2.27	-0.83, 1.23	Normal (0.38, 1.01), truncated between -0.83 and 1.23	[8]

from feces to hides at processing plant							
$MD_{hc\_BR\_hide\_carc}$ - Transfer factor of BR-EC concentration from hides pre-dehiding to carcass pre-evisceration	1.72	0.67	0.876	1.05, 2.39	-0.53, 3.98	0.77, 2.60	Normal (1.72, 1.15), truncated between 0.77 and 2.60

[a] Minimum and maximum effect sizes reported in primary studies included in the MA

[b] Lower and upper limits of the 95% prediction interval of the pooled effect size

[c] Minimum of lower limits of 95% confidence intervals and maximum of upper limits of 95 confidence interval across primary studies in the MA

[d] Lognorm2 (μ, σ) represented the lognormal distribution with specified mean and standard deviation generated from the "logged" values of the distribution. Truncation was conducted by discarding the values exceeding the restricted range and re-allocating the "lost" probability proportionally across the remaining range between min. and max.

Text S1. Fitting the odds ratio (*OR*) and logarithmic mean difference (*MD*) to lognormal and normal distributions based on the results from the meta-analysis (MA)

To quantify the effect of a particular processing step on the contamination of *E. coli* of beef cattle, odds ratio (*OR*) and logarithmic mean difference (*MD*, $\log_{10}$ CFU scale) were introduced to measure the changes in the prevalence and concentration between before and after the processing step, respectively. The term of odds was defined as the ratio of the probability of *E. coli*-positive samples to the probability of *E. coli*-negative samples, and was expressed as $P/(1 - P)$, in which P referred to the prevalence, i.e., the proportion of samples being positive for resistant *E. coli* of the total samples being tested in this case. The *OR* represented the ratio of the odds of being resistant *E. coli* positive after a particular processing step, to the odds of the outcome before the processing step. The equations of *OR* and *MD* are listed below:

$$OR = \frac{P_{i+1} \times (1 - P_i)}{P_i \times (1 - P_{i+1})} \quad \text{Equation S1}$$

where P_i and P_{i+1} are the prevalence (%) before and after a particular processing step;

$$MD = C_i - C_{i+1} \quad \text{Equation S2}$$

where C_i and C_{i+1} are the concentrations ($\log_{10}$ CFU) before and after a particular processing step.

Based on the relationship, the post-prevalence/concentration were predicted given the estimates of *OR*/*MD* and pre-prevalence/concentration, as follows.

$$P_{i+1} = \frac{OR \times P_i}{1 - P_i + OR \times P_i} \quad \text{Equation S3}$$

$$C_{i+1} = C_i - MD \quad \text{Equation S4}$$

Instead of using the reported prevalence or concentration data from a single empirical study to calculate the *OR* or *MD* using Equation S1 or S2, MA approach was used to estimate the effects of various processing steps on the contamination changes in *E. coli* by fitting reported effect sizes from multiple relevant primary studies via random-effects model, considering both between- and within-study variance [83].

The relevant primary studies were identified through comprehensive literature reviews (CLR). Briefly, the procedure of CLR can be summarized as: first, the research question of our interests was proposed as "what is the impact of commercial processing steps on the population changes in contamination of β-lactam resistant *E. coli* or other *E. coli* strains in cattle"; second, the search strategy was determined with three main concepts regarding the research question: *E. coli*, cattle, and decontamination/intervention/processing; third, the searching keywords and syntax relevant to the research question were developed based on the selected bibliographic databases, including PubMed and Web of Science Core Collection; last, the screening of relevance was performed according to pre-structured inclusion-exclusion criteria. The primary studies were considered relevant and included in MA if information pertinent to the prevalence and/or concentration changes of *E. coli* due to a particular cattle processing step in the farm-to-abattoir continuum was reported. Summary statistics and relevant information, particularly the number of *E. coli*-positive samples, the sample size, mean concentration in $\log_{10}$ CFU scale, standard deviation/error and 95% confidence interval of concentration, and sampling season, were manually extracted, organized and stored in a Microsoft Excel® 2013 spreadsheet (Microsoft Corp., Redmond, WA). In addition, antibiotic resistance profile of *E. coli* (β-lactam resistant or generic) was also collected, regardless of pathogenicity. Other biobibliographical information, such as first author, year of publication, and geographical location were collected as the identification of eligible studies. Due to the large number variables that required CLR for data collection, swift literature reviews were conducted to fulfill the purpose of model development. Different from a typical systematic review process, only one reviewer conducted the step of relevance screening and data extraction/verification. Whenever a question was raised by the primary evidence reviewer,

discussion with a senior author was made for make a final decision. Studies included in each MA can be find in **Table S10** and **Figures S1-S10**.

With raw data extracted from published primary studies, MA was conducted to fit the distributions of *OR* and *MD* using random-effects model [84], assuming that the true log *OR* and *MD* followed normal distributions (μ, σ^2). Random-effects MA was executed in R 3.4.0 using the "metafor" package [85]. The parameter of μ was reported as the "average" true effect estimate of the aggregate log *OR* or *MD*, and the variance (σ^2) around the point estimate was calculated as $se^2 + \tau^2$ reported in the outputs. Here τ^2 models the between-study variation. However, using τ^2 itself as an estimator of σ^2 may underestimate the variability of the true log *OR* or *MD* of new studies, because it does not account for the uncertainty in estimating μ, which is se^2. Mathematically, se^2 is largely driven by within-study variation on σ^2 due to sampling error. Details about the estimation results are summarized in **Table S10** and displayed in the form of forest plots (**Figures S1-S10**). In our QMEA model, the change in *E. coli* concentration (*MD*) was estimated separately for generic and β-lactam resistant *E. coli*, assuming the great difference in naturally-occurring microbial loads between generic and resistant *E. coli* may largely influence the change in microbial load due to a specific processing step.

Outputs related to heterogeneity estimates and tests in MA showed significant heterogeneity commonly existed for the log *OR* and *MD* estimates across primary studies. Heterogeneity was quantified by I^2-statistic representing the amount of total variation across studies caused by heterogeneity rather than chance, and tested by Cochrane's *Q*-statistic [86]. Heterogeneity with I^2 between 75% and 100 % might be considerable important, while it might not be important with I^2 between 0 % and 40 % [83]. Except for $L\_OR_{hh\_farm\_plant}$ with $I^2 < 40\%$, all the other MA variables in our study were with an I^2 showing significant heterogeneity. The great heterogeneity across studies can be a result of the difference in antibiotic administration, processing conditions, sampling design, microbiological testing and other factors that are likely to occur in reality and lead to the naturally-occurring variation in effects on microbial contamination among different cattle primary production and processing facilities. Hence, CLR and MA enable more representative estimates of log *OR* and *MD* to capture the naturally-occurring variation.

Our applications of CLR and MA allow for generating the distributions of log *OR* and *MD* by taking into consideration the "full" variation at a great extent. However, it is also equally important to avoid the extreme values in the distributions that are unlikely to occur in reality, as the impossible values may bias QMEA final output estimates. Truncation techniques were applied to set the boundaries of distributions to rule out extreme values. The truncation boundaries were determined as the lower and upper limits of the 95% confidence intervals of primary studies included in the random-effects MA, which can be found from **Figures S1-S10** and **Table S10**. This truncation approach allows for a wide range for capturing all the observations reported in the primary studies included in MA. In addition, it even covers a wider range than the 95% prediction interval of the aggregate effect size, indicating a strong capability to capture the possible underlying effect in a new study that is similar to but covered in the MA.

Text S2. Fitting the prevalence of BR-EC in RWA feces at the feedlot ($P_{f\_BR\_RWA}$) to a beta-binomial mixture distribution using a hierarchical model

To evaluate the impact of antimicrobial use on the occurrence of BR-EC in cattle production system, a CLR was conducted to collect data to compare the presence of BR-EC in the feces of beef cattle raised in CONV vs RWA farms. Based on data obtained from this CLR, the impact factor of BR-EC prevalence between RWA and CONV feces (*IF*) was estimated. As the referent group for estimating *IF*, prevalence of BR-EC in the feces of RWA cattle reported in multiple primary studies were retrieved. *IF* and BR-EC prevalence in RWA cattle feces were incorporated in the QMEA model as input variable, based on which, BR-EC prevalence in CONV cattle can be estimated. The estimation of distribution parameters of *IF* was described in **Table S10**. The estimation of input variable distribution for the prevalence of BR-EC in RWA feces is described here.

Critical statistics, including sampling month, the number of positive samples, and the sample size, were extracted from eligible studies, and then stored in Microsoft Excel® 2013 spreadsheets. Other identification information of the eligible studies, such as the first author, year of publication, country was extracted as well. Previous studies have shown a great seasonal impact on the BR-EC shedding in cattle [16]. Hence, sampling months were categorized into

high shedding season (June to September) and low shedding season (October to May). Studies lacking information about the sampling season were assumed to have a 50%-50% chance of the two shedding periods.

The unobserved true prevalence conditional on sampling seasons was assumed to follow a beta distribution with two parameters determining the shape of distribution. Distribution shape parameters were obtained by fitting the extracted data to the beta-binomial mixture model using maximum likelihood estimation. Therefore, the outcome of $P_{f\_BR\_RWA}$ was expressed as two beta distributions, one modelled the prevalence in high shedding season, and the other one was for that in low shedding season. For samples with unknown sampling season, a 0.5-0.5 mixture of the two beta distributions was assumed. Note that these models are for the unobserved "true prevalence", not the observed sample prevalence. The shape parameters generated by R 3.4.0 were 0.41 and 1.07 for high shedding season ($H\_P_{f\_BR\_RWA}$), and 1.29 and 2.00 for low shedding seasons ($L\_P_{f\_BR\_RWA}$), respectively.

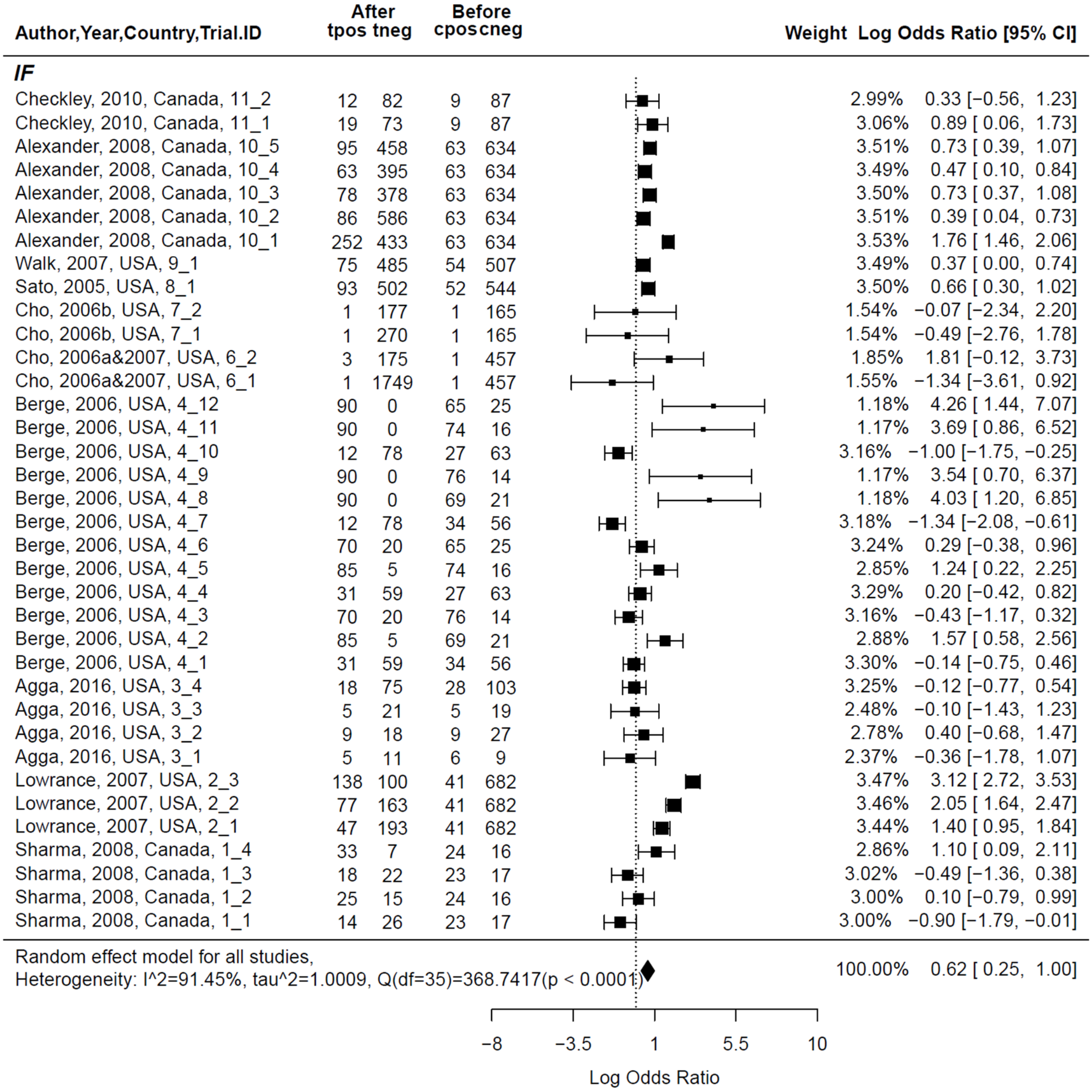

Figure S1. Forest plot for eligible studies used to fit the impact factor of BR-EC prevalence between RWA and CONV feces (*IF*).

Note: tpos/tneg - the number of positive/negative samples in CONV group; cpos/cneg- the number of positive/negative samples in RWA group.

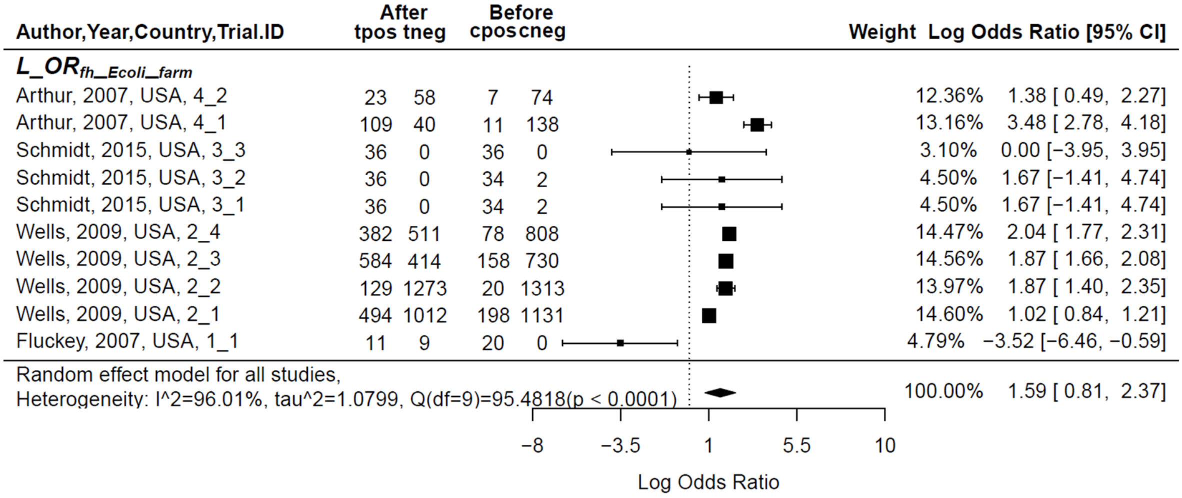

Figure S2. Forest plot for eligible studies used to fit the transfer ratio of *E. coli* prevalence from feces to hides at the feedlot in the low-shedding season ($L\_OR_{fh\_Ecoli\_farm}$).

Note: tpos/tneg - the number of positive/negative samples in hides group; cpos/cneg- the number of positive/negative samples in feces group.

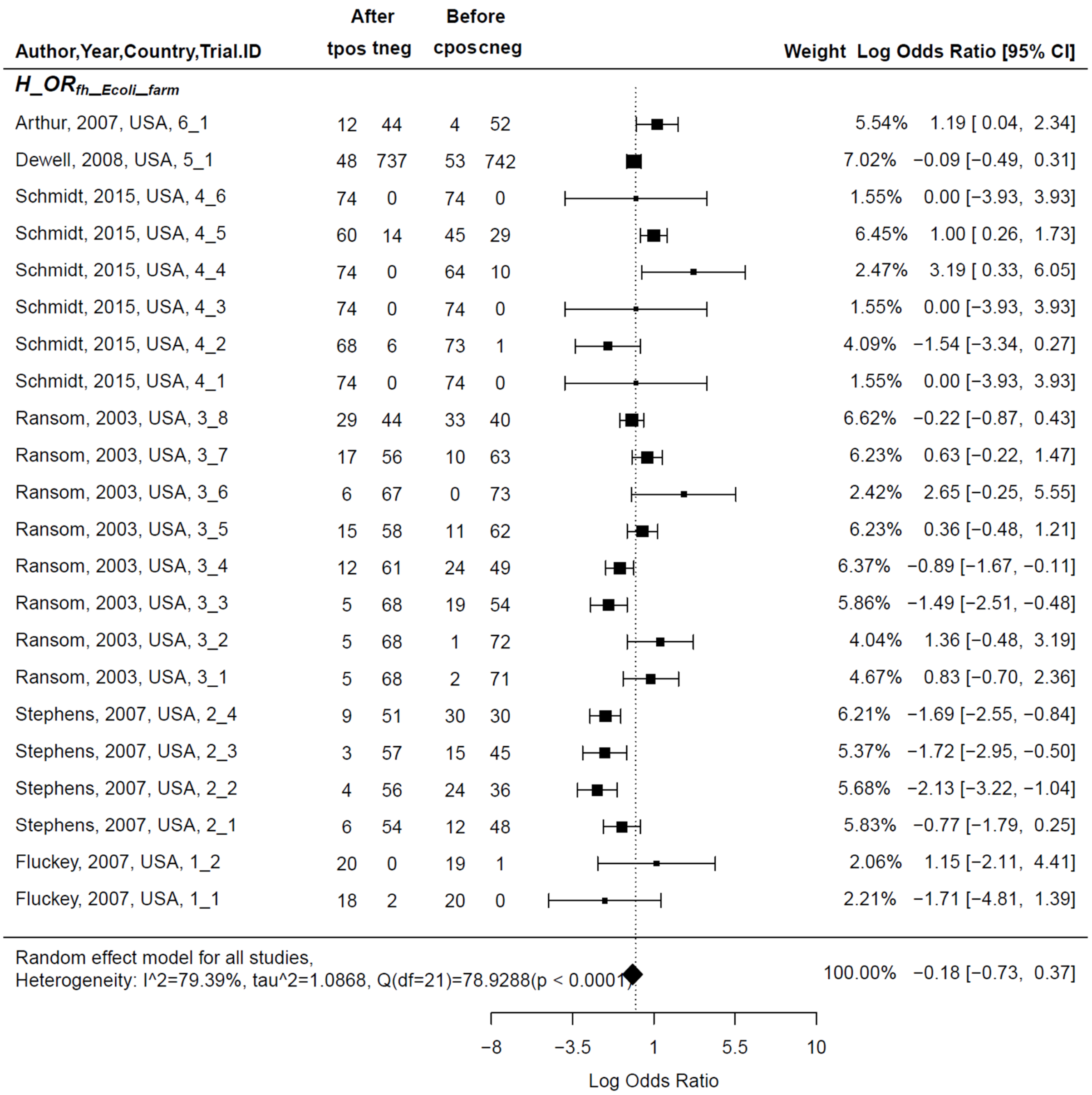

Figure S3. Forest plot for eligible studies used to fit transfer ratio of *E. coli* prevalence from feces to hides at the feedlot in the highshedding season ($H\_OR_{fh\_Ecoli\_farm}$).

Note: tpos/tneg - the number of positive/negative samples in hides group; cpos/cneg- the number of positive/negative samples in feces group.

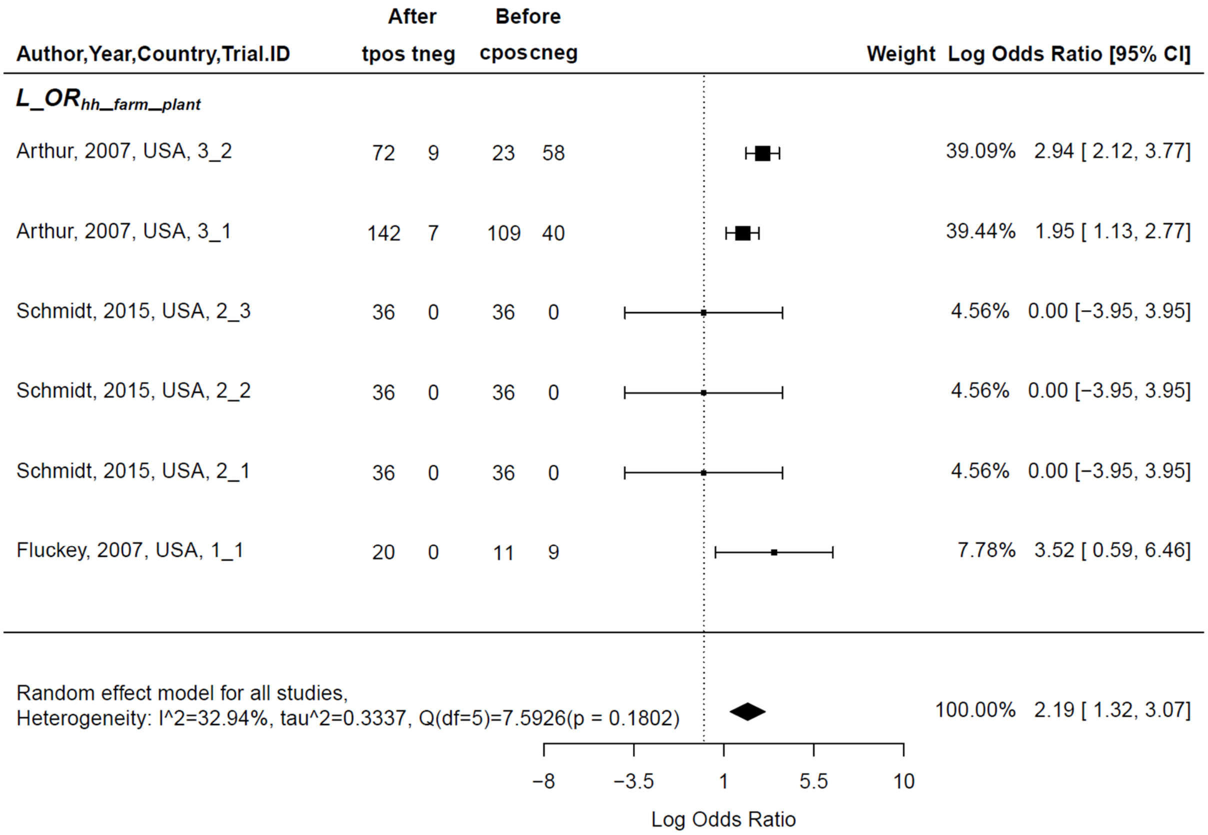

Figure S4. Forest plot for eligible studies used to fit the transfer ratio of *E. coli* prevalence from hides at the feedlot to hides sampled immediately before dehiding in the low-shedding season ($L\_OR_{hh\_farm\_plant}$).

Note: tpos/tneg - the number of positive/negative samples in pre-dehiding hides group; cpos/cneg- the number of positive/negative samples in feedlot hides group.

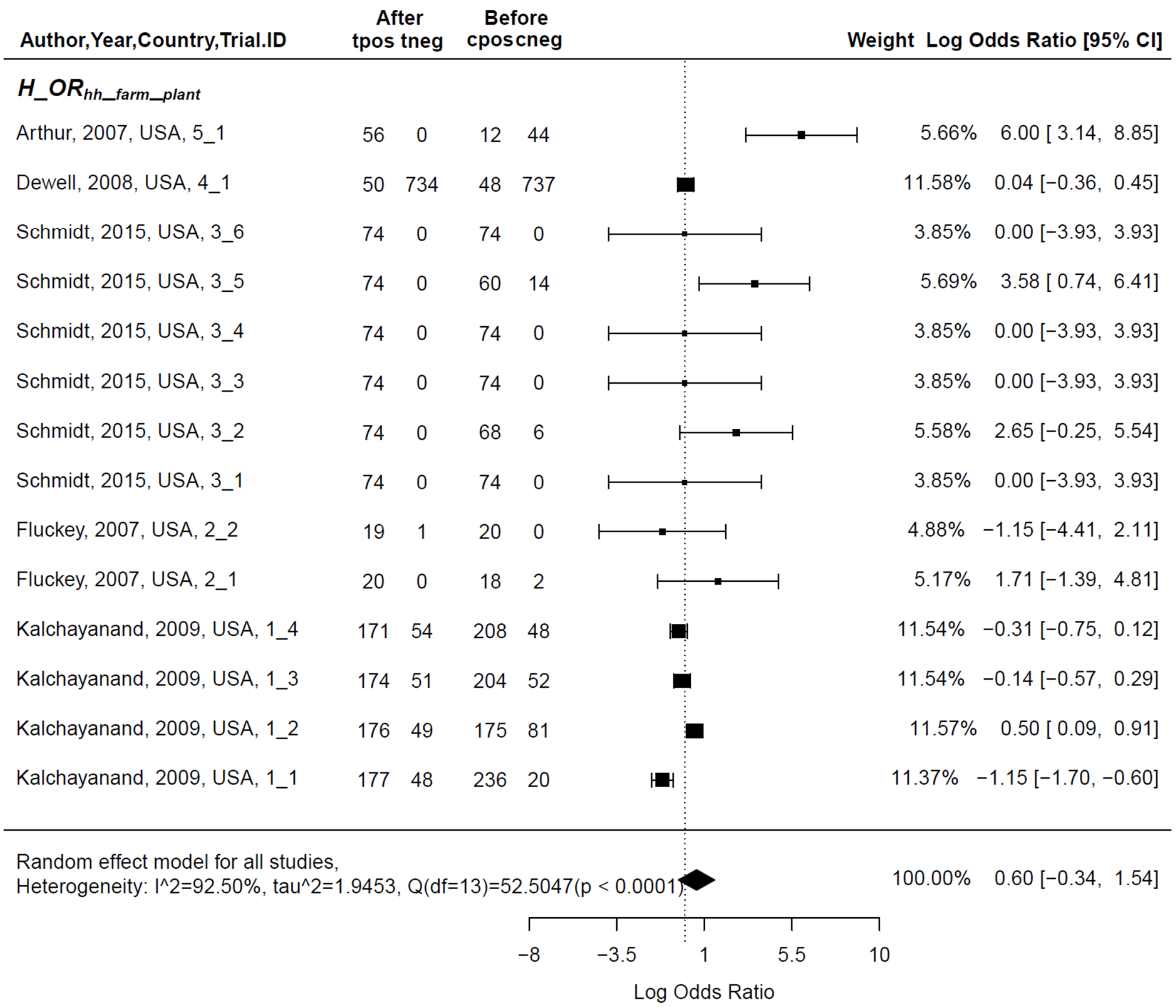

Figure S5. Forest plot for eligible studies used to fit transfer ratio of *E. coli* prevalence from hides at the feedlot to hides sampled immediately before dehiding in the high-shedding season ($H\_OR_{hh\_farm\_plant}$).

Note: tpos/tneg - the number of positive/negative samples in pre-dehiding hides group; cpos/cneg- the number of positive/negative samples in feedlot hides group.

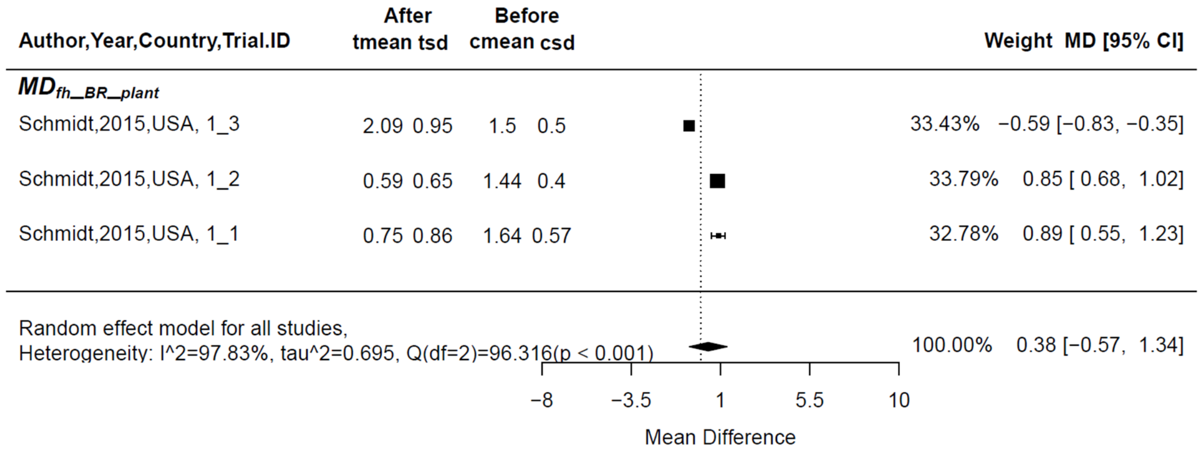

Figure S6. Forest plot for eligible studies used to fit the transfer factor of BR-EC concentration from feces to hides at the processing plant ($MD_{fh\_BR\_plant}$).

Note: tmean/tsd - the average concentration/standard deviation in hides group; cmean/csd - the average concentration/standard deviation in feces group.

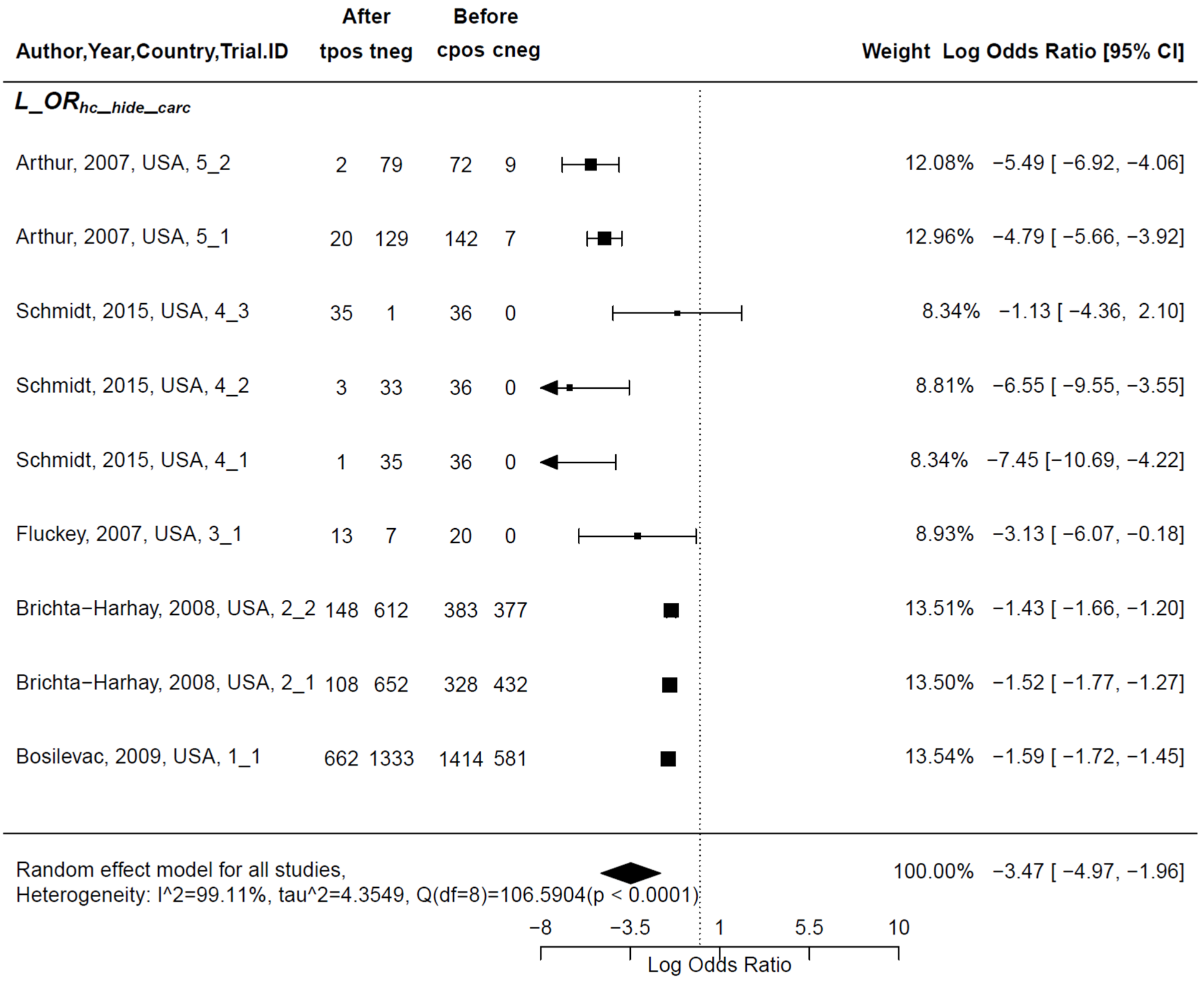

Figure S7. Forest plot for eligible studies used to fit the transfer ratio of *E. coli* prevalence from the hide pre-dehiding to the carcass pre-evisceration in the low-shedding season ($L\_OR_{hc\_hide\_carc}$).
Note: tpos/tneg - the number of positive/negative samples in pre-evisceration carcass group; cpos/cneg- the number of positive/negative samples in pre-dehiding hides group.

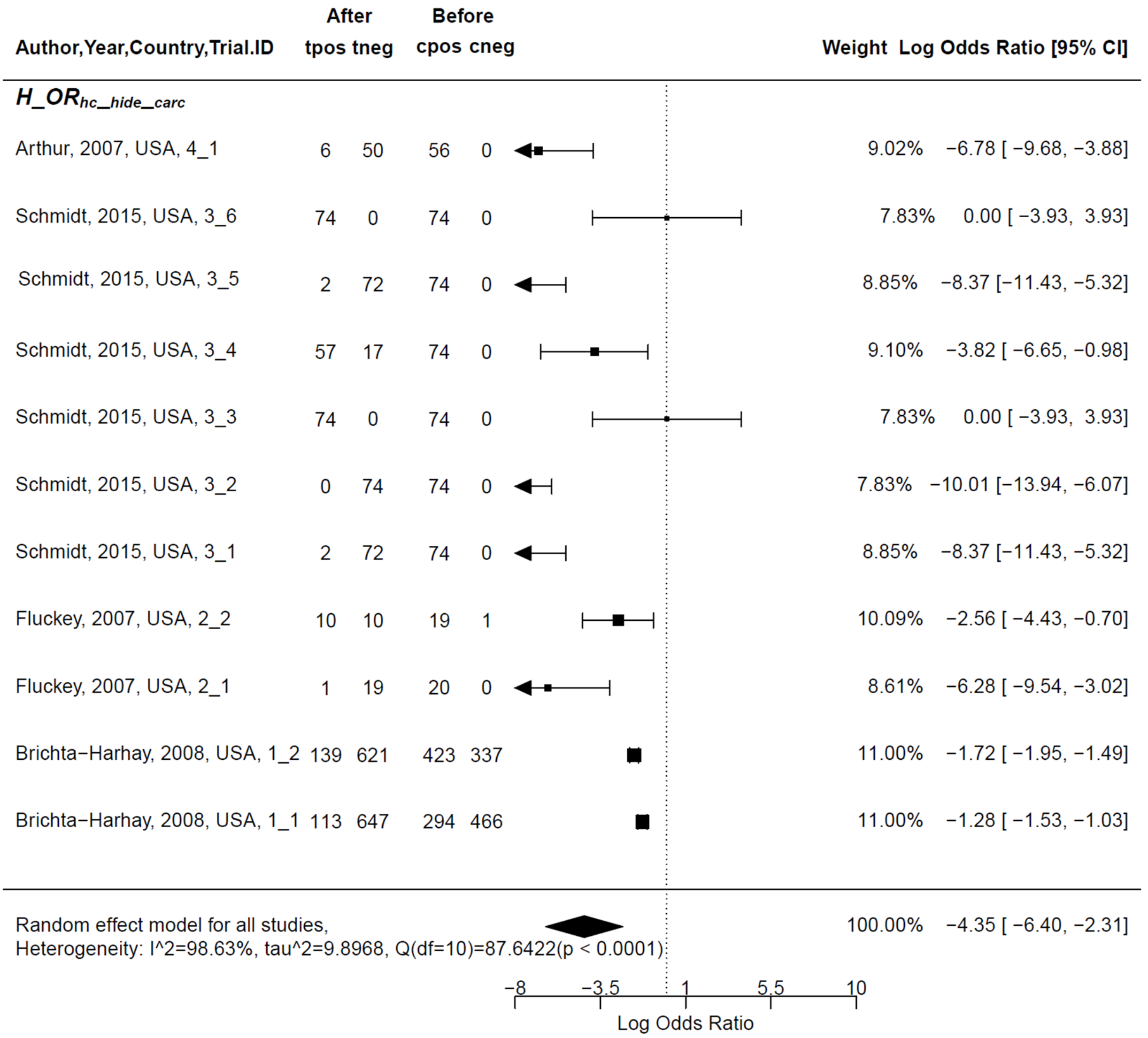

Figure S8. Forest plot for eligible studies used to fit the transfer ratio of *E. coli* prevalence from the hide pre-dehiding to the carcass pre-evisceration in the high-shedding season ($H\_OR_{hc\_hide\_carc}$).

Note: tpos/tneg - the number of positive/negative samples in pre-evisceration carcass group; cpos/cneg- the number of positive/negative samples in pre-dehiding hides group.

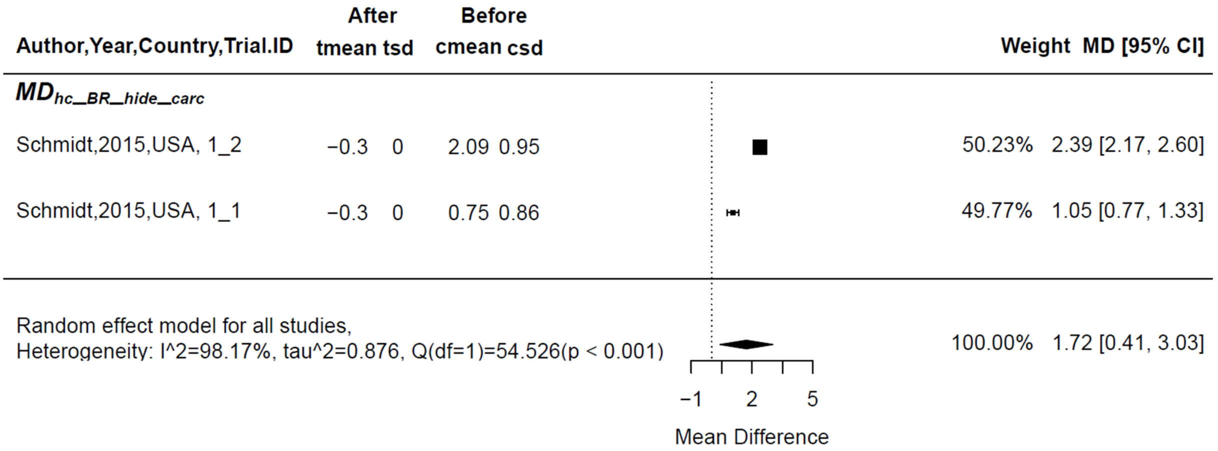

Figure S9. Forest plot for eligible studies used to fit the transfer factor of BR-EC concentration from the hide pre-dehiding to the carcass pre-evisceration ($MD_{hc\_BR\_hide\_carc}$).

Note: tmean/tsd - the average concentration/standard deviation in pre-evisceration carcass group; cmean/csd - the average concentration/standard deviation in pre-dehiding hides group.

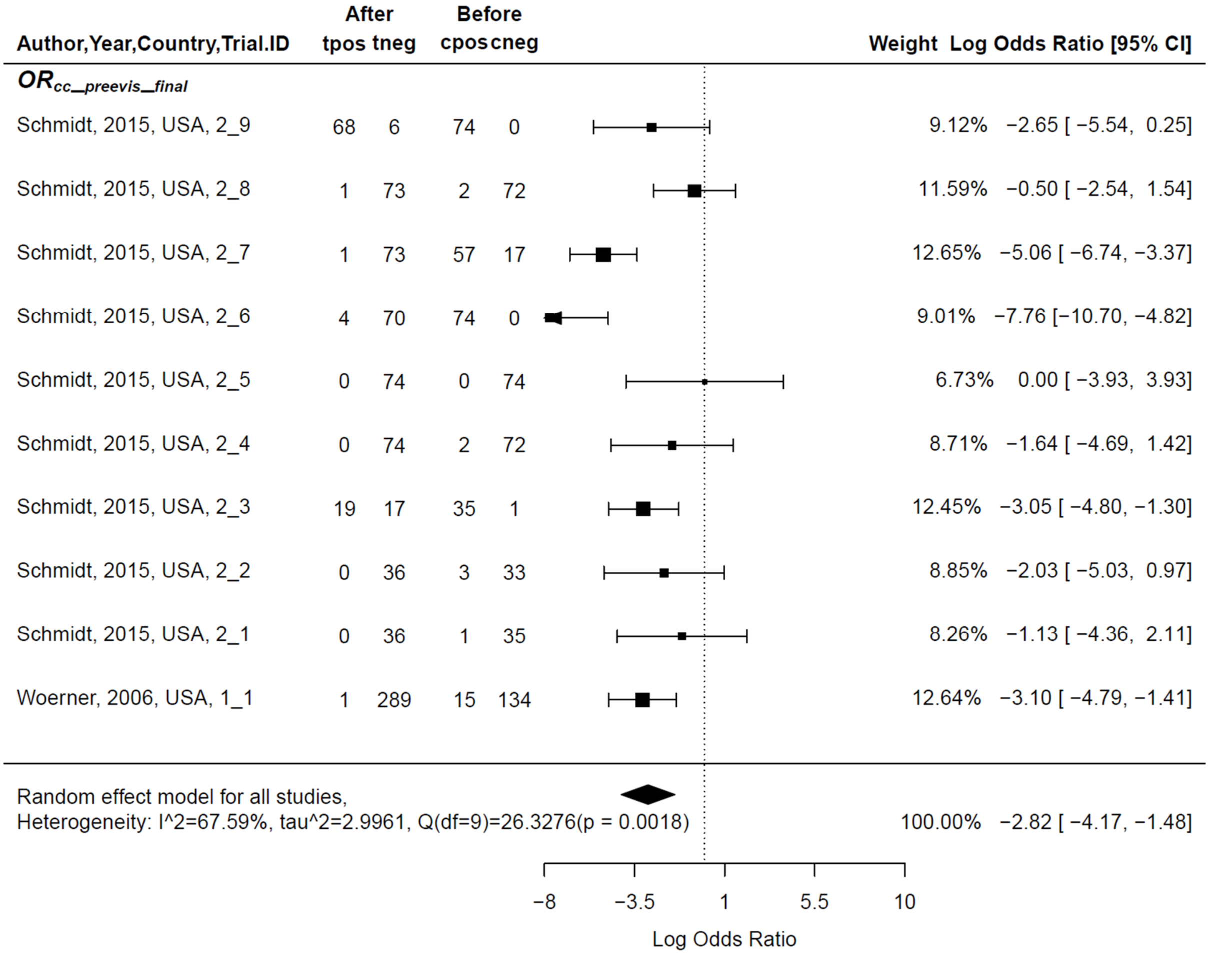

Figure S10. Forest plot for eligible studies used to fit the transfer ratio of *E. coli* prevalence due to evisceration ($OR_{cc\_preevis\_final}$).

Note: tpos/tneg - the number of positive/negative samples in final carcass group; cpos/cneg- the number of positive/negative samples in pre-evisceration carcass group.
